# Comprehensive insight into endothelial progenitor cell-derived extracellular vesicles as a promising candidate for disease treatment

**DOI:** 10.1186/s13287-022-02921-0

**Published:** 2022-06-07

**Authors:** Ke Chen, Yang Li, Luwei Xu, Yiguan Qian, Ning Liu, Changcheng Zhou, Jingyu Liu, Liuhua Zhou, Zheng Xu, Ruipeng Jia, Yu-Zheng Ge

**Affiliations:** Department of Urology, Nanjing First Hospital, Nanjing Medical University, No. 68 Changle Road, Nanjing, 210006 Jiangsu People’s Republic of China

**Keywords:** Endothelial progenitor cells, Extracellular vesicles, Disease treatment, Cell communication, Comprehensive review

## Abstract

Endothelial progenitor cells (EPCs), which are a type of stem cell, have been found to have strong angiogenic and tissue repair capabilities. Extracellular vesicles (EVs) contain many effective components, such as cellular proteins, microRNAs, messenger RNAs, and long noncoding RNAs, and can be secreted by different cell types. The functions of EVs depend mainly on their parent cells. Many researchers have conducted functional studies of EPC-derived EVs (EPC-EVs) and showed that they exhibit therapeutic effects on many diseases, such as cardiovascular disease, acute kidney injury, acute lung injury, and sepsis. In this review article, we comprehensively summarized the biogenesis and functions of EPCs and EVs and the potent role of EPC-EVs in the treatment of various diseases. Furthermore, the current problems and future prospects have been discussed, and further studies are needed to compare the therapeutic effects of EVs derived from various stem cells, which will contribute to the accelerated translation of these applications in a clinical setting.

**Supplementary Information:**

The online version contains supplementary material available at 10.1186/s13287-022-02921-0.

## Introduction

In recent years, many research studies have revealed the different roles of stem cells and proposed their applications in the treatment of various diseases. Among the various classes of stem cells, which mainly include embryonic, haematopoietic, mesenchymal, and neural types, one class named endothelial progenitor cells (EPCs) has specifically attracted interest. Initially discovered by Asahara et al. in 1997, EPCs can be recruited to ischaemic tissue sites where they enhance collateral vessel growth [1]. EPCs can differentiate into mature endothelial cells (ECs) and directly participate in angiogenesis and revascularization [2]. Further studies have revealed that EPCs are involved in the repair and regeneration of damaged tissues [3–5]. Specifically, EPCs were found to be involved in the regeneration of ischaemic organs, playing important roles in the treatment of ischaemic brain injury [6] and repair of ischaemic renal tissue [7]. Additionally, the results from animal experiments and clinical studies have revealed that EPC-mediated therapy alleviates pulmonary arterial hypertension [8].

Extracellular vesicles (EVs), which are small intraluminal vesicles derived from different types of cells, are involved in the transport of endocellular contents such as cellular proteins, microRNAs (miRNAs), messenger RNA (mRNA), and long noncoding RNAs (lncRNAs) to the cell exterior [9]. EVs were previously described as “garbage bags” used by mature reticular cells to dispose of transferrin receptors [10], but their potential as carriers of intercellular communication is gradually being revealed [11]. Originating from bone marrow (BM), EPCs circulate in the peripheral blood (PB) and then migrate to the sites of both pathological and physiological angiogenesis [12]. EPC transplants were shown to stimulate angiogenesis by triggering angiogenic events or by differentiating into mature ECs [2]. However, EPC transplantation has some disadvantages, such as the potential for immunogenicity, malignant transformation, and embolus formation [13]. Most cell types, including EPCs, can release EVs [14]. Recent lines of evidence have demonstrated that stem cells likely achieve their effects through exosome secretion [15]. Some researchers have focused on EPC-derived EVs (EPC-EVs) because they are easier to manipulate than EPCs and have a stronger effect on skin wound healing and angiogenesis [16]. Moreover, due to the specific biological structure and features of these cells, anaphylactic reactions to EPC-EVs and their rejection by the body are seldom reported [17]. In this review article, we summarize the relationships between EPC-EVs and various diseases and then discuss current problems and future prospects related to their use for disease treatment.

## Biogenesis and function of EPCs

EPCs are mainly derived from BM, PB, or cord blood (CB) and usually selected on the basis of certain cell surface antigen markers, such as vascular endothelial growth factor receptor 2 (VEGFR-2), CD34, and CD133 [18]. Additionally, culture and colony assays can be used to isolate EPCs [19]. These cells are usually classified as early or late EPCs according to their biological properties and culture time. Early EPCs appear after 5–7 days of culture and have a low proliferation rate, whereas late EPCs derived from mononuclear cells appear after 14–21 days of culture and have a high proliferative potential [20].

**Fig. 1:**
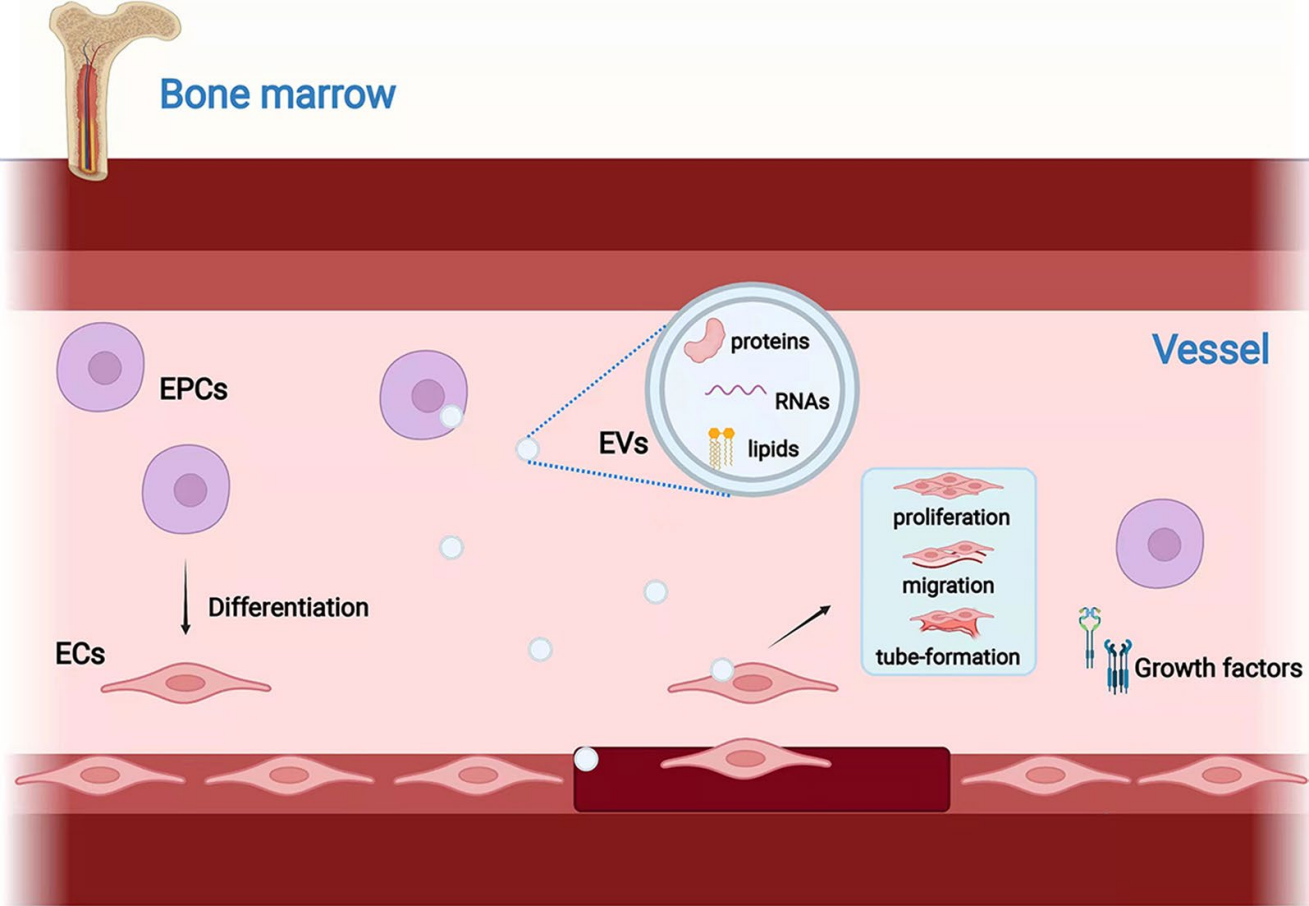
Biogenesis and function of EPCs. Bone marrow-derived EPCs can differentiate into ECs; or stimulate the proliferation, migration, and tube formation of ECs through release of EVs, which contain proteins, RNAs, and lipids. (Created with BioRender.com)

As presented in Fig. 1, EPCs repair damaged vessel walls through four steps: mobilization, homing, invasion, and differentiation/paracrine effects [21]. First, ischaemia promotes the transcription of molecules related to angiogenesis, such as adhesion molecules on ECs and vascular endothelial growth factor (VEGF), the latter of which is known to mobilize EPCs for differentiation [22]. Injured vasculature also induces EPC mobilization via other cytokines [23]. Once in the bloodstream, EPCs are affected by the concentration of chemokines and home to the vascular injury site. Upon reaching the site where the chemotactic agent is produced, the EPCs attach to the activated ECs of the damaged blood vessel wall via adhesion molecules and then enter the extracellular matrix from the vascular lumen via the endothelial intima, where they undergo differentiation into ECs. Additionally, EPCs can initiate vascular repair through paracrine signalling [24].

## Biology of EVs

### Biogenesis and release of EVs

**Fig. 2:**
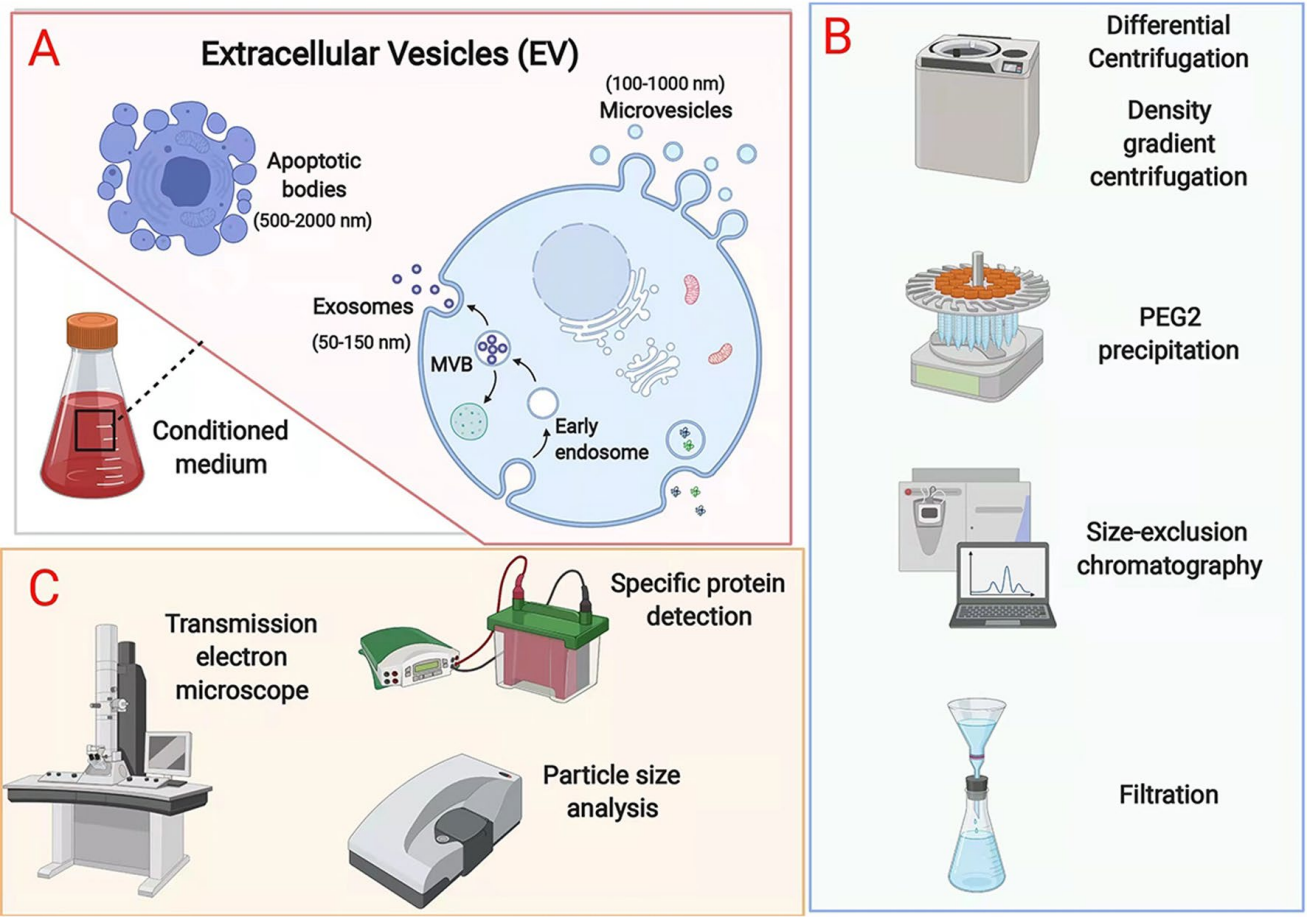
Isolation and characterization of EVs. **A** EVs have three subtypes: apoptotic bodies (500–2,000 nm), exosomes (50–150 nm), and microvesicles (100–1000 nm). **B** Isolation methods. **C** Characterization of EVs. (Created with BioRender.com)

EVs are produced in the form of apoptotic bodies (500–2,000 nm in diameter), exosomes (50–150 nm), and microvesicles (100–1,000 nm) [25], with the exosomes being distinguished from the other two EV types on the basis of surface protein expression and mode of biogenesis [26] (Fig. 2A). EVs are characterized by specific markers, such as membrane proteins (CD9, CD63, and CD81), major histocompatibility complex (MHC), Alix, TSG101, and HSP70 [27]. Exosomes also contain other proteins, DNA, and different types of RNA including miRNAs, mRNAs, and lncRNAs [28]. The processes of EV formation are complicated. In general, early endosomes are formed by cell membrane invagination, after which they will transform into late endosomes. Multivesicular bodies containing intraluminal vesicles are formed by inward budding of the limiting membrane, whereupon some are transported for cargo degradation in the lysosomes, some are transferred to the trans-Golgi network, and some release their intraluminal vesicles extracellularly as exosomes through fusion with the plasma membrane [25, 28, 29]. Target cells absorb EVs by endocytosis and fusion. Receptors on the membrane can facilitate EV uptake and regulate the cell signalling pathway [28, 30]. Although microvesicles have higher densities than exosomes, both types of EVs may overlap in size [31]. Microvesicles are formed through outward budding and fission of the plasma membrane and then shed [32]. Apoptotic bodies, the largest of the three types of EVs, contain nuclear material, organelles, membranes, and cytosolic content. Apoptotic bodies are released during the late stage of cell death through the membrane blebbing of apoptotic cells, a process induced through the phosphorylation of myosin light chain by Rho-associated kinase 1 (ROCK1), which in turn is activated by caspase-3 [33–35].

### Isolation and characterization of EVs

EVs can be isolated by several different methods (Fig. 2B). Differential centrifugation is a classic strategy. EVs are separated by gradually increasing centrifugation time and centrifugal force according to the different sedimentation rates of protein molecules, cells, and cell fragments in uniform suspensions [36]. Density gradient centrifugation is based on density differences in sample components, causing them to move to different locations [37]. The most widely used method is ultracentrifugation, which is relatively rapid, has a high efficiency, and results in a high purity [38]. However, there is no standard method for isolating EVs by centrifugation. PEG can also be used to change the solubility of EVs for precipitation because it can bind to the hydrophobic lipid bilayer due to its size and properties [39]. This method has the advantages of few devices needed, a high yield rate, and simple operation, but the purity of the EVs is low [40]. EVs can be differentiated on the basis of size, such as with ultrafiltration and size exclusion chromatography (SEC). The former method results in an unsatisfactory purity [41], while the latter method is time-consuming [42]. Having been widely used to separate biopolymers, gel filtration can be applied to isolate EVs [43], but this method is limited because it requires pretreatment to concentrate the EV samples [44]. Other approaches, such as methods involving lectins, antibodies, and lipid-binding proteins, use intermolecular interactions to capture EVs. Although their products are highly pure, these materials are expensive and difficult to process in large quantities [45].

As shown in Fig. 2C, transmission electron microscopy (TEM) and scanning electron microscopy can evaluate the morphology and structure of EVs [46]. Since the sizes of most of them are smaller than the minimum optical resolution, electron microscopes are the only way to visualize EVs [47]. Nanoparticle tracking analysis (NTA) can measure the particle concentration and size distribution [48] and occasionally the zeta potential [49]. In most studies, Western blotting (WB) is adopted to detect the surface markers of EVs [50] as WB can reveal the presence and amount of target proteins [51].

### Potential biological functions of EVs

Emerging evidence has shown that EVs can act as biomarkers and therapeutic targets for various diseases. Additionally, these molecules play a vital role in cell communication.

#### Biomarker function

EVs are detectable in various bodily fluids, such as urine [52], blood [53], breast milk [54], and saliva [55]. They carry specific molecules from the parental cells and can also reflect current disease status. For example, microvesicles derived from human nipple aspirate fluid and blood are considered sources of nonintrusive molecular biomarkers for the early detection of various cancer types [56]. In the development of methods for EV detection, researchers have demonstrated that target membrane proteins can be used. Logozzi et al. was the first to develop a new enzyme-linked immunosorbent assay for detecting Rab-5b/caveolin-1 double-positive EVs in melanoma patients [57]. Shao et al. developed a rapid and sensitive analytical microfluidic chip platform that can distinguish patients with glioblastoma multiforme from healthy individuals [58]. The same research group also developed a high-throughput screening method that distinguishes ovarian cancer patients with a high degree of accuracy by targeting epithelial cell adhesion molecule (EpCAM) and CD24 on EVs in ascites [59]. Overall, EV-associated proteins can be used for disease detection, as they are more likely to be cancer related.

RNA is another important molecule found within EVs. Valadi and colleagues were the first to propose that mRNA- and miRNA-containing EVs might exert specific functions in recipient cells [11]. According to Ogata-Kawamata et al., colorectal cancer patients have high levels of seven miRNAs, which are decreased after tumour resection [60]. Matsumura et al. suggested that miR-19a-3p carried in EVs could serve as a prognostic biomarker to predict the recurrence of colorectal cancer [61]. The detection of EV-related RNAs identifies a novel biomarker strategy for cancer diagnosis and prognosis.

#### Cell communication

**Fig. 3:**
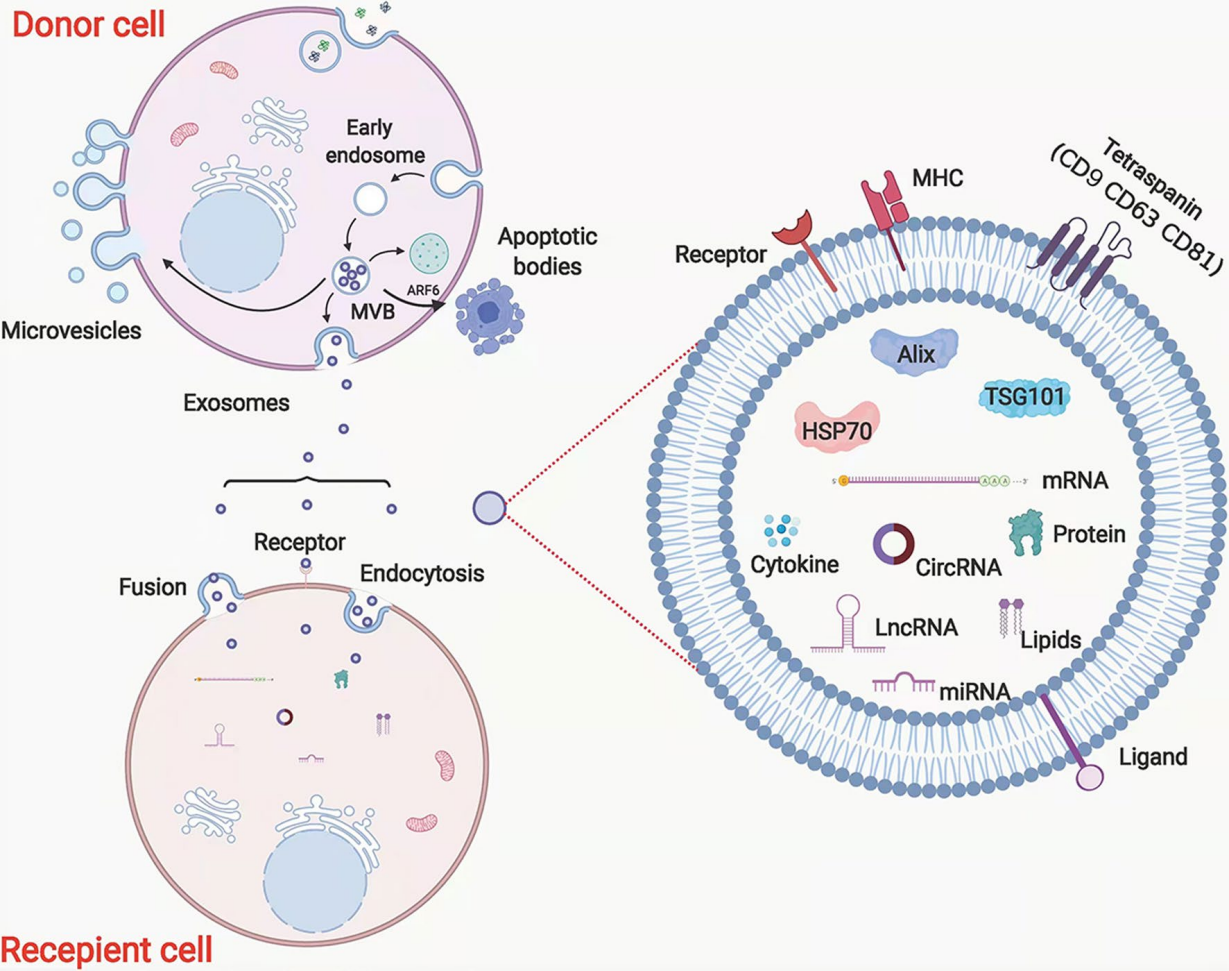
EVs function as cell communication. Target cells absorb EVs by endocytosis and fusion, while receptors on the membrane can facilitate EVs uptake and regulate cell signal pathway. EVs are characterized by specific markers, such as membrane proteins (CD9, CD63, CD81), major histocompatibility complex (MHC), Alix, TSG101, and HSP70. (Created with BioRender.com)

Accumulating lines of evidence have indicated that cell communication occurs through paracrine and endocrine signalling pathways (Fig. 3). Raposo et al. was the first to discover that antigen-specific T cell responses were induced by B cell-derived EVs with functional MHC II peptide complexes [62]. Samuelson et al. summarized the effect of EVs on cell-to-cell communication in metabolic regulation [63], and a similar role in exercise-induced adaptations was also reported [64]. According to Capra et al., EVs play important roles in various cell communication aspects, such as embryo–maternal crosstalk and oocyte maturation, fertilization, and implantation [65].

In cancer patients, EVs were shown to mediate tumour-to-stroma, stroma-to-tumour, and tumour-to-tumour communication [66]. EVs secreted by tumours can modulate the tumour microenvironment [66], thereby establishing cell communication pathways to support tumorigenesis [67]. The induction of tumour-promoting stroma by tumour-related EVs has been demonstrated [68] in prostate cancer, osteosarcoma, breast cancer, and colorectal cancer cells [69–72]. Conversely, stroma-secreted EVs can promote the growth, invasion, and metastasis of tumour cells. For example, Richards et al. demonstrated that EVs derived from cancer-associated fibroblasts enhanced the Snail protein level in pancreatic ductal adenocarcinoma cells, promoting their proliferation and drug resistance [73]. The EV-mediated transfer of cancer cell-derived signals may also exert vital functions in different tumour cell subpopulations. For example, Al-Nedawi and colleagues showed that glioblastoma-derived EVs transferring the oncogenic EGFRvIII receptor led to oncogenic signalling activation in recipient tumour cells [74].

#### Therapeutic targets

To date, three types of EV-targeting therapeutic strategies have been proposed [75]. One of these is to eliminate the secretion of EVs, given that the molecules they carry can disturb cancer therapeutics and contribute to tumour progression, as reported for human epidermal growth factor receptor 2 (HER-2) located on EVs [76]. In this regard, Marleau et al. have already developed a system that can target HER-2 to bind cancer cell-derived EVs [77]. Chen et al. discovered that programmed death-ligand 1 (PD-L1), which acts in concert with programmed cell death protein 1 (PD-1) to suppress the antitumor immune response, was enriched in cancer-derived EVs and thus proposed the use of exosomal PD-L1 as a novel predictive biomarker for anti-PD-1 therapy [78]. Additionally, circulating EVs can be inhibited. For example, the neutral sphingomyelinase inhibitor GW4869 is widely used to inhibit both the release of EVs and the formation of intraluminal vesicles [79]. Blocking EV uptake is another way to suppress tumour progression, as shown in a study that used proteinase K to decrease the absorption of EVs by cancer cells [80]. All these studies demonstrated that EV-targeted therapy may be a new strategy for cancer treatment.

## Potential roles of EPC-EVs in the treatment of various diseases

EPCs are widely considered a possible cell therapy source to promote tissue repair. However, the use of EPCs presents some concerns, such as ethical issues, cellular rejection, infusion toxicity, ectopic tissue formation, and possible tumorigenicity [81]. Therefore, EPC-EVs have emerged as novel alternatives other than EPCs. As summarized in Additional file 1: Table S1 and Additional file 2: Table S2, EPC-EVs carry multiple common exosome markers, such as CD9, CD63, and CD81 [82], and some EPC-specific molecules including VEGFR-2, CD133, and CD34. Since the initial discovery of EPC-EVs, they have exhibited better therapeutic efficacy for various ischaemic diseases than traditional treatments. With continuing research, EPC-EVs have been shown to have various therapeutic roles in other diseases. In the following sections, we discuss the potent role of EPC-EVs in the treatment of various diseases and the current problems in their application as well as future perspectives (Additional file 1: Table S1).

### Role in treatment of kidney disease

Under most circumstances, acute kidney injury (AKI) is caused by ischaemia–reperfusion injury (IRI) [83]. Recent studies, including ours, have shown that renal IRI can be repaired by EPCs through differentiation into ECs or endocrine/paracrine pathways [84–90]. Given these findings, Cantaluppi et al. postulated that EPC-EVs can exert a protective effect in AKI [91]. They found that EPC-EVs were localized within tubular cells and peritubular capillaries when injected following ischaemia–reperfusion. This process enhanced tubular cell proliferation and reduced leukocyte infiltration and cell apoptosis, which in turn conferred morphological and functional protection against AKI. In addition, EPC-EVs prevented the progression of chronic kidney damage by inhibiting glomerulosclerosis, capillary thinning, and tubulointerstitial fibrosis. Mechanistically, the renoprotective effect of EPC-EVs was exerted via transferring miR-126 and miR-296. AKI can also be induced by sepsis [92], a serious condition caused by a dysfunctional inflammatory response to infection [93]. Using a mouse model of sepsis-induced AKI mediated by lipopolysaccharide (LPS) treatment, He et al. found that EPC-EVs could inhibit apoptosis and inflammation by transferring miR-93-5p. In addition, miR-93-5p was found to directly target and inhibit KDM6B, induce H3K27me3, and inhibit TNF-α activation, thereby attenuating cell injury [94]. In another mouse model of sepsis-induced AKI constructed with caecal ligation and puncture (CLP) treatment, EPC-EVs were demonstrated to alleviate sepsis-induced AKI by releasing miR-21-5p to silence Runt-related transcription factor 1 (RUNX1). In the in vivo experiments, the researchers found reduced tubular degeneration and monocyte infiltration in the rats treated with miR-21-5p [95].

Glomerulonephritis, an inflammatory disease that affects the filtration of the glomeruli, can cause progressive fibrotic damage and chronic renal failure [96]. Cantaluppi et al. discovered that EPC-EVs could reduce proteinuria, suppress mesangial cell activation, leucocyte infiltration, and apoptosis, and inhibit glomerular injury by transporting specific RNAs. However, they did not determine the effective component of EPC-EVs [97]. In a follow-up study, these researchers demonstrated that EPC-EVs could protect the integrity of the glomerular filtration barrier from cytokine- and complement-induced injury, indicating that they may have a role in glomerulonephritis treatment [98]. Using a mouse model, Yang et al. showed that EPC-EVs could decrease pericyte–myofibroblast transition in renal fibrosis, thereby attenuating the injury [99].

### Role in treatment of lung disease

Acute lung injury (ALI) is characterized by the infiltration of neutrophils in the alveolar–capillary barrier [100]. During ALI, inflammation causes sustained injury to the capillary endothelial barrier, resulting in pulmonary oedema, increased pulmonary vascular permeability, and hypoxemia [101]. With an ALI animal model generated via intratracheal administration of LPS, Wu et al. observed that histopathological ALI changes were significantly weakened, shown as reduced interstitial oedema, bleeding, alveolar wall thickness, and neutrophil infiltration in the lung parenchyma and alveolar space, and the arterial blood PaO_2_ was improved in the group treated with EPC-EVs [102]. Through further in vitro experiments, the authors found that the knockdown of miR-126 inhibited the phosphorylation of RAF and extracellular signal-regulated kinases 1/2 (ERK1/2), whereas the EPC-EV transfer of this miRNA into target cells led to the downregulation of Sprouty-related Ena/vasodilator-stimulated phosphoprotein homology-1 domain 1 (SPRED1) expression and promotion of the RAF/ERK1/2 signalling pathways, which subsequently improved the function of ECs [102]. Similarly, using a combination of next-generation sequencing, an ALI mouse model, and in vitro transfection assays, Zhou et al. proved that EPC-EVs containing miR-126 could reduce the damage of ALI, whereas NIH3T3 cell-derived EVs carrying little miR-126 could not [103].

### Role in treatment of bone disease

Long-bone defects are common conditions presented by patients in orthopaedic departments. For patients with long-bone defects attributed to postsurgical infections and complications or trauma, distraction osteogenesis (DO) is the primary treatment option [104]. Although DO can induce neo-osteogenesis, it requires a long consolidation period and has an increased risk of subsequent complications [105]. Jia et al. evaluated the effect of EPC-EVs in a rat model of unilateral tibial DO; bone regeneration was strongly accelerated, as shown by histological, X-ray, and micro-computed tomography analyses in the EPC-EVs-treated group [106]. Additionally, the EPC-EV group had a higher vessel density than the control group. The results demonstrated that EPC-EVs strengthened EC migration, proliferation, and angiogenesis in a miR-126-dependent manner. Qin and Zhang observed that BM stromal cells treated with EPC-EVs showed decreased calcium deposition but increased colony-forming unit fibroblasts. The results indicated that EPC-EVs suppressed the expression of osteogenic genes and increased the proliferation of BM stromal cells, thereby regulating their osteoblastic differentiation in vitro [107]. Cui and colleagues proved that lncRNA-MALAT1 contained in EPC-EVs could promote bone repair by enhancing the recruitment and differentiation of osteoclast precursors [108].

Degenerative osteoporosis is usually a common issue in the elderly. However, the incidence of steroid-induced osteoporosis (SIOP) among younger individuals has shown an increasing trend [109]. There remain no effective treatment options for SIOP to date, simple and feasible therapies for this condition are urgently needed. Lu et al. established a mouse model of SIOP using high-dose dexamethasone and demonstrated through histopathological analysis that EPC-EVs treatment could increase the density and volume of the BM and trabecular bone [110]. Using Kyoto Encyclopedia of Genes and Genomes (KEGG) mapping, the authors further found that EPC-EVs partly reversed injury-induced changes in the ferroptosis pathway. Moreover, EPC-EVs reduced the dexamethasone-induced alterations in several oxidative injury markers.

### Role in treatment of neurological injury

Stroke, a common ischaemic disease, is caused by the accumulation of inflammatory cells and the release of inflammatory factors due to local vascular tissue hypoxia and ischaemia, leading to local vascular EC necrosis and apoptosis [111]. Ischaemic stroke occurs when blood flow to the brain is disrupted. Restoration of flow or reperfusion can reduce injury but must be performed very early after ischaemia occurs [112]. Reactive oxygen species (ROS) and nitrogen are produced in the ischaemic penumbra during ischaemia and reperfusion [113]. Although ROS-induced vascular EC injury is known to play an important role in IRI, effective strategies to resolve this condition are lacking [114]. Wang et al. studied the relationship between hypoxia–reoxygenation (HR) injury and EPC-EVs in human brain microvascular ECs (hbECs). The authors concluded that EPC-EVs elicited their effects by regulating ROS production and the phosphatidylinositol 3-kinase (PI3K)/endothelial nitric oxide synthase (eNOS)/nitric oxide (NO) pathway. They also found that caspase-3 and miR-126 were delivered to hbECs by EPC-EVs. Unfortunately, the researchers did not further explore the underlying mechanism [115]. Similarly, Ma et al. overexpressed miR-210 in EPC-EVs, which resulted in significant reductions in HR-induced angiogenic dysfunction, EC apoptosis, and ROS production. However, these researchers could not rule out the influence of downstream functional targets, such as Efan3, Ptp1b, ISCU, and COX10 [116]. Li et al. found that miR-137 had neuroprotective effects against mitochondrial dysfunction and apoptosis, which might be dependent on the miR-137-cyclooxygenase 2 (COX2)/prostaglandin E2 (PGE2) signalling pathway [117].

Amyotrophic lateral sclerosis (ALS) is a serious neurological disease that can also cause cardiovascular failure [118]. Garbuzova-Davis et al. transplanted EPCs intravenously into a symptomatic superoxide dismutase 1 (SOD1)^G93A^ mouse model of ALS to replace their damaged ECs, successfully restoring the blood–brain barrier [119, 120]. Subsequently, the same research group demonstrated that EPC-EVs could reduce mouse brain EC damage, identifying a new cell-free treatment for endothelial repair in ALS [121]. However, whether the protective effects of EVs on damaged cells are durable and whether cellular damage is reversible remain unclear. Furthermore, these in vitro results require EV administration to a mouse model of ALS in vivo to confirm the endothelial repair effect of the EPC-EVs.

### Role in treatment of myocardial infarction

Myocardial infarction (MI) is one of the main causes of mortality and morbidity worldwide [122]. Cardiac fibroblasts are crucial for cardiac cell proliferation, angiogenesis, and cardiac tissue homeostasis and remodelling [123]. Ke et al. discovered that human EPC-EVs could increase angiogenesis and proliferation in cardiac fibroblasts by decreasing the expression of high mobility group box 1 protein B1 (HMGB1) and promoting mesenchymal–endothelial transition [124]. Further research proved that treatment with Exo-miR-363-3p or Exo-miR-218-5p improved the MI induced by left coronary artery ligation and recovered the integrity of the myocardial tissue. Compared with that of the control group, collagen expression was downregulated, and the degree of myocardial fibrosis was reduced in the EPC-EVs group. However, there are still some problems to be solved, such as the localization of miRNAs in EPC-EVs and identification of more potential miRNAs. Moreover, the role of EPC-EVs in the IRI model should be explored in the future, as the animal model in this study was a non-IRI model [125]. Huang et al. manipulated EPC-EVs with miR-1246 or miR-1290 and found that the upregulated expression of either miRNA could promote phenotypic changes of fibroblasts to ECs and angiogenesis in cardiac fibroblasts, whereas their downregulation produced the opposite effects. However, the phenotype changes should be interpreted with caution as more markers are needed to characterize fibroblasts and ECs [126]. Yue et al. used IL-10-knockout (KO) mice to mimic inflammation and then compared the protein levels and therapeutic effect of exosomes derived from IL-10-KO-EPCs and wild-type EPCs (WT-EPCs). WT-EPC-Exo treatment strongly suppressed cell apoptosis, decreased MI scar size, improved left ventricular cardiac function, and facilitated post-MI neovascularization, whereas IL-10-KO-EPC-Exo treatment produced the opposite effects [127]. Liu et al. isolated circulating exosomes from mice with streptozotocin-induced diabetes for use in in vivo and in vitro experiments and concluded that exosomal miR-144-3p may hinder the mobilization of EPCs, which was related to the neovascularization damage induced by ischaemia, suggesting a novel strategy to improve cardiac repair after MI by intervening in the enriched miR-144-3p [128].

Some studies have demonstrated the consistency of the therapeutic effect of cardiac transplant cells without engraftment, indicating that paracrine mechanisms could potentially be used for therapeutic effects [129, 130]. Chen et al. delivered EPC-EVs within a shear-thinning gel to facilitate their exact localization and continuous delivery and succeeded in reproducing the advantageous effects of EPC treatment. In vivo studies showed that the delivery of EVs within the shear-thinning gel led to preservation of the ventricular geometry, enhanced peri-infarct vascular proliferation, and improved haemodynamic function after MI, thereby increasing the EV-mediated myocardial preservation effect. Nevertheless, translating shear-thinning gel as an EV-delivered tool to treat acute MI still requires more animal studies to test this approach [131]. Similarly, Chung et al. used a shear-thinning gel to deliver EPC-EVs and proved that such delivery at 4 days after MI preserved the holistic ventricular geometry and improved left ventricular contractility [132].

### Role in treatment of non-MI cardiovascular injury

The incidence of cardiovascular disease continues to increase annually worldwide. Vascular endothelial injury may cause potential changes, such as thrombosis, inflammation, and smooth muscle cell (SMC) proliferation, leading to neointimal hyperplasia, unfavourable arterial remodelling, and restenosis [133]. Using a rat model of vascular injury, Li et al. found that EPC-EVs accelerated re-endothelialization at an early stage after injury, and in vitro analyses indicated that this treatment could strengthen the proliferation and migration of ECs [134]. SMC proliferation is believed to be a key factor for restenosis following endothelial injury [135]. In a study by Kong et al. on the effects of exosomes on SMCs and ECs, in vivo assays showed that the intimal-to-medial area ratio was significantly reduced and SMC proliferation was significantly lower in the exosome group than in the control group [136]. The in vitro study confirmed that the administration of exosomes could significantly enhance the migration and proliferation of SMCs and ECs, indicating that EPC-EVs likely inhibited neointimal hyperplasia in the rat model through the promotion of EC repair. Hu et al. proved that EPC-EVs were more efficacious than EC-EVs for vascular repair [137]. In another study, Hu et al. investigated the mechanism of EPC-EVs in endothelial repair and concluded that the exosomal delivery of miR-21-5p may promote EC repair by inhibiting thrombospondin 1 (THBS1). However, the researchers could not rule out the effects of other EPC-EVs in EC repair. Therefore, further experiments are required to rule out the interference of other miRNAs [138]. Recently, EPCs expressing the bone matrix protein osteocalcin were found to be related to the severity of cardiovascular diseases [139]. Subsequently, Yi et al. proved that overexpressed osteocalcin (OCN) in EPCs had beneficial effects on EC proliferation, migration, and function through the exosomal pathway, participating in the promotion of angiogenesis and NO formation via the interaction of OCN and its receptor G protein-coupled receptor family C group 6 member A (GPRC6A). Due to the lack of specific receptor antagonist of GPRC6A and the fact that OCN and GPRC6A are not specific to each other, the findings should be verified with further well-designed studies [140]. Cardiovascular homeostasis is regulated by the renin–angiotensin system, in which angiotensin II (Ang II) is the main peptide and is related to vascular dysfunction [141]. Wang et al. suggested that EPCs could repair EC injury through their exosomal effects on mitochondrial function and angiotensin-converting enzyme 2 (ACE2) overexpression [142]. These researchers subsequently proved that ACE2 enhanced the effects of EPC-EVs on the Ang II-induced inhibition of vascular SMC phenotypic modulation by restraining nuclear factor-kappa B (NF-κB) signalling [143].

Cardiovascular diseases can be caused by many other diseases, such as bronchopulmonary dysplasia (BPD), a severe lung disease in extremely preterm infants [144]. Using an experimental model of BPD obtained by exposing pulmonary microvascular ECs to hyperoxia, Zhang et al. proved that the administration of EPC-EVs enhanced the bioactivity of ECs in vitro and increased the expression levels of VEGF, VEGFR-2, and eNOS relative to those in the untreated hyperoxia group. However, the exact carriers of EPC-EVs and the molecular mechanisms remain yet to be explored, and the in vivo studies are warranted to validate the ex vivo findings [145]. Atherosclerosis is a chronic inflammatory disorder characterized by endothelial dysfunction. Li et al. proved that EPC-EVs can suppress the ferroptosis of ECs and mitigate the occurrence of atherosclerosis by transferring miR-199a-3p to inhibit specificity protein 1 (SP1) [146].

### Role in treatment of sepsis

Sepsis, which is a dysfunctional systemic inflammatory disease caused by infection, usually leads to organ failure and even death [147]. Previous studies have shown that EPCs have beneficial effects on organ dysfunction, vascular injury, and mortality in sepsis models [148]. Based on the assumption that EPC-EVs can transfer miRNAs to protect the microvasculature, Zhou et al. used the CLP method to generate a mouse model of sepsis to test their hypothesis, and EPC-EVs treatment improved the survival rate of septic mice, inhibited renal and lung vascular leakage, and reduced kidney and liver dysfunction. The sepsis-induced increase in plasma cytokine and chemokine levels was also attenuated by EPC-EVs. The investigators explored the genome-wide miRNA expression patterns in EPC-EVs and focused on one specific miRNA: miR-126. However, they could not rule out the influence of additional miRNAs, lipids, and proteins [149]. Similarly, Hong and colleagues discovered that EPC-EVs improved heart function by suppressing oxidative stress, inflammation, and apoptosis and attenuated the pathological damage of myocardial tissues in septic rats, providing a novel therapeutic strategy against myocardial damage in sepsis [150].

According to a whole-blood transcriptomic study, lncRNA taurine upregulated gene 1 (TUG1) is one of the top five sepsis-relevant lncRNAs [151]. TUG1 alleviated sepsis-induced inflammation and apoptosis by targeting growth factor receptor-bound protein 2 (GRB2)-associated binding protein 1 (GAB1) and miR-34b-5p [152]. Ma et al. explored the effect of EPC-EVs-delivered TUG1 in septic mice and demonstrated that miR-9-5p could bind competitively with TUG1, which upregulated the expression of sirtuin 1 (SIRT1) and promoted M2 macrophage polarization. In a mouse model, EPC-EVs carrying TUG1 were shown to reduce the organ damage induced by sepsis mainly through macrophage M2 polarization [153].

### Role in treatment of diabetes

The global incidence of diabetes is increasing, and complications have become serious public health problems [154]. Approximately 20% of patients develop diabetic wounds, the most common being leg or foot ulcers [155], which can reduce physical activity, result in chronic ischaemic skin lesions, and even lead to limb amputations in serious cases [156]. Li et al. found that EPC-EVs could heal diabetic wounds in diabetic rats and enhance the proliferation, migration, and tube formation of vascular ECs in vitro. Moreover, ECs stimulated with these exosomes showed increased expression of angiogenesis-related molecules. However, this study focused on the phenotype assays without exploring the active ingredients in EPC-EVs [157]. Similarly, Zhang et al. discovered that the proliferation, migration, and tube formation of ECs could be enhanced by EPC-EVs. Furthermore, the exosomal treatment altered the expression of genes related to the ERK1/2 signalling pathway, which was the pivotal mediator during the EV-induced angiogenic responses of ECs by functional study confirmation [158]. Xu et al. treated diabetic mice bearing skin wounds with miR-221-3p, EPC-EVs, or phosphate-buffered saline and showed that wound healing was strengthened by EPC-EVs and miR-221-3p compared to that in both control and diabetic mice. In this study, EPC-EVs were directly spread onto wound sites, which was convenient and practical in the clinical settings [159].

Patients living with diabetes have a 3–4 times higher risk of suffering an ischaemic stroke than those without diabetes because the combination of decreased angiogenesis and impaired endothelial dysfunction aggravates cerebral damage [160]. Wang found that EPC-EVs protected ECs against HR-induced dysfunction and injury [115] and enhanced the function and viability of EPCs in diabetes [161]. In a subsequent study, these researchers found that EPC-EVs–miR-126 had better effects than EPC-EVs in increasing cerebral blood flow and microvascular density, decreasing the infarct size, and promoting neurogenesis and angiogenesis as well as neurological functional recovery. However, as different cell types including neurons, vascular ECs, and neuroblasts can express VEGFR-2, further research is needed to determine which brain cells are the main ones with increased VEGFR-2 expression [162]. Atherosclerosis is a main macrovascular complication of diabetes and the leading cause of death in patients with diabetes [163]. Bai et al. found that most of the top ten upregulated miRNAs in EPCs-derived exosomes were associated with atherosclerosis. Further in vivo studies showed that treatment with EPC-EVs could reduce the production of inflammatory factors and diabetic atherosclerotic plaques. Nonetheless, the exact miRNA in EPC-EVs that plays an important role in diabetic atherosclerosis remains unclear [164].

## Future perspectives and conclusions

With the development of regenerative medicine and stem cell therapy, various stem cells have been proven to be effective in tissue damage repair [165, 166]. EPCs are one class of stem cells that are known to repair and regenerate various tissues [81, 167] and have attracted increasing attention for their stem cell-specific functions as well as the regulatory function of their EVs. EPC-EVs have exhibited better effects than EPCs in some preclinical studies [131]. EVs are less immunogenic than their parental cells and can be transported and stored for a long time [168]. Moreover, aside from their abilities to deliver various molecules that regulate angiogenesis, fibrosis, and cell proliferation, EPC-EVs can be utilized as cell-free drug carrier systems, which have the advantages of availability and reproducibility [169]. The feasibility of loading or enriching EVs with specific regulatory molecules has been proven [170, 171], and the application of modified EPC-EVs as vehicles for delivering regulatory factors may elicit better therapeutic effects.

However, some issues should be addressed before the translational application of EPC-EVs in the clinical settings. Firstly, the differential therapeutic effects of EVs derived from various stem cells including EPCs remain to be determined. To date, various classes of stem cells and their EVs were applied in disease treatment, among which adipose-derived stem cells (ADSCs) and bone marrow stem cells (BMSCs) were studied extensively [172–175]. As summarized in Additional file 2: Table S2, the EVs from ADSCs and BMSCs exhibited similar characteristics and exerted protective functions in a wide array of diseases like EPC-EVs. Recently, Miyasaki and colleagues compared the efficiency of EVs from mesenchymal stem cells and EPCs in treating chronic kidney disease and found similar improvement in renal function in terms of serum albumin, cystatin C, crystals in the renal tubules, and fibrosis, rather than staining of alpha-smooth muscle actin [176]. However, the direct comparison between different types of EVs was rare, and further studies are needed.

Secondly, a consensus on the source, identification, and culture conditions of EPCs is needed. As stated before, EPC-EVs isolated from PB [91], CB [94], and BM [177] exhibited protective effect on kidney injury, through different mechanisms. The number of EPCs in BM (0.05%) and PB (0.01%) is relatively low [178], while CB-derived EPCs have a higher proliferative potential and higher frequency than other EPC types [179, 180]. Although positivity for CD133, VEGFR-2, and CD34 is widely accepted when referring to EPCs, two distinct classes (early or late EPCs) were reported with different combinations of defining markers, which needs standard protocols for the detection of EPCs [19]. Regardless of the cell source and identification process, the number of EPCs was too low for further applications, while ex vivo culture can contribute to the matureness of stem cells and a reduction in cell number [178]. Hence, the head-to-head comparative studies on EVs derived from EPCs with different cell source, definition, and culture conditions should be performed, which might conclude a consensus and guideline for the clinical application.

Thirdly, the exact effective molecules within EPC-EVs remain to be explored comprehensively. Most of the cited studies focused on the phenotype changes under EPC-EVs treatment, which lacked validation from different levels and detailed investigation of the specific active ingredients in EPC-EVs. Different components have been reported in EPC-EVs, such as miRNAs, lncRNAs, proteins (Additional file 1: Table S1), and mRNAs [181]. Among which, miRNAs have been explored widely, and most of the candidate molecule was selected without high-throughput screening. Mounting evidence has indicated the existence of lipids [182], circRNAs [183], and DNA [184] in the EVs derived from different stem cells, which remain yet unknown in EPC-EVs. Further well-designed studies with substantial verification are warranted to examine the effective components in a comprehensive and unbiased manner.

Lastly, the EV extraction process is complicated and has yet to be standardized. The quality standard of the entire production process must be medically satisfied and certified, which affects economic sustainability [185]. Importantly, the mass production of EPC-EVs is critical for clinical needs [186]. Moreover, the establishment of the dose and delivery route of EPC-EVs requires more research, as the amount and content of exosomal cargo are different under different pathophysiological conditions. Most studies simply analysed the amount and content of exosomes at a single time point and did not completely investigate all exosomal contents that were differentially expressed.

Because the complexity and high off-target rate of EVs are the main obstacles to clinical application, a new strategy for comprehensively simulating EVs is needed [187]. Although artificial EVs are easier to mass-produce and more uniform [188], additional research is needed in the preclinical setting. Hopefully, with the continued development of tissue engineering and nanotechnology, transporting EPC-EVs to the right place can be achieved, which may result in more effective treatment of different diseases. Given our limited understanding of EPC-EVs, their long-term therapeutic safety is difficult to predict. Therefore, extra care is required during the transition of this treatment strategy to the clinic.

## Supplementary Information

**Additional file1: Table S1.** The beneficial effects of EPC-EVs on various diseases.

**Additional file2: Table S2.** The beneficial effects of ADSC- and BMSC-EVs on various diseases.

## Data Availability

Not applicable.

## Abbreviations

ADSCs: Adipose-derived stem cells
Ang II: Angiotensin II
ACE2: Angiotensin-converting enzyme 2
ALI: Acute lung injury
ALS: Amyotrophic lateral sclerosis
AKI: Acute kidney injury
BM: Bone marrow
BMSCs: Bone marrow stem cells
BPD: Bronchopulmonary dysplasia
CB: Cord blood
CLP: Caecal ligation and puncture
COX2: Cyclooxygenase 2
DO: Distraction osteogenesis
ECs: Endothelial cells
eNOS: Endothelial nitric oxide synthase
EpCAM: Epithelial cell adhesion molecule
EPC-EVs: EPC-derived EVs
EPCs: Endothelial progenitor cells
ERK1/2: Extracellular signal-regulated kinases 1/2
EVs: Extracellular vesicles
GPRC6A: G protein-coupled receptor family C group 6 member A
GAB1: Growth factor receptor-bound protein 2 (GRB2)-associated binding protein 1
GRB2: Growth factor receptor-bound protein 2
hbECs: Human brain microvascular ECs
HER-2: Human epidermal growth factor receptor 2
HMGB1: High mobility group box 1 protein B1
HR: Hypoxia reoxygenation
IRI: Ischaemia–reperfusion injury
KEGG: Kyoto Encyclopedia of Genes and Genomes
KO: Knockout
lncRNAs: Long noncoding RNAs
LPS: Lipopolysaccharide
MiRNAs: MicroRNAs
mRNA: Messenger RNA
MI: Myocardial infarction
MHC: Major histocompatibility complex
NTA: Nanoparticle tracking analysis
NO: Nitric oxide
NF-κB: Nuclear factor kappa B
OCN: Osteocalcin
PB: Peripheral blood
PD-L1: Programmed death-ligand 1
PD-1: Programmed cell death protein 1
PI3K: Phosphatidylinositol 3-kinase
PGE2: Prostaglandin E2
ROCK1: Rho-associated kinase 1
ROS: Reactive oxygen species
RUNX1: Runt-related transcription factor 1
SEC: Size exclusion chromatography
SPRED1: Stimulated phosphoprotein homology-1 domain 1
SIOP: Steroid-induced osteoporosis
SOD1: Symptomatic superoxide dismutase 1
SP1: Specificity protein 1
SIRT1: Sirtuin 1
SMC: Smooth muscle cell
TEM: Transmission electron microscopy
THBS1: Thrombospondin 1
TUG1: Taurine upregulated gene 1
VEGF: Vascular endothelial growth factor
VEGFR-2: Vascular endothelial growth factor receptor 2
WB: Western blotting
WT-EPC: Wild-type EPCs

## References

[1] Asahara T, Murohara T, Sullivan A, Silver M, van der Zee R, Li T, Witzenbichler B, Schatteman G, Isner JM (1997). Isolation of putative progenitor endothelial cells for angiogenesis. Science.

[2] Critser PJ, Yoder MC (2010). Endothelial colony-forming cell role in neoangiogenesis and tissue repair. Curr Opin Organ Transplant.

[3] Ratajczak J, Kucia M, Mierzejewska K, Marlicz W, Pietrzkowski Z, Wojakowski W, Greco NJ, Tendera M, Ratajczak MZ (2013). Paracrine proangiopoietic effects of human umbilical cord blood-derived purified CD133+ cells–implications for stem cell therapies in regenerative medicine. Stem Cells Dev.

[4] Zhao F, Zhou L, Xu Z, Xu L, Xu Z, Ping W, Liu J, Zhou C, Wang M, Jia R (2020). Hypoxia-preconditioned adipose-derived endothelial progenitor cells promote bladder augmentation. Tissue Eng Part A.

[5] Zhou L, Xia J, Qiu X, Wang P, Jia R, Chen Y, Yang B, Dai Y (2015). In vitro evaluation of endothelial progenitor cells from adipose tissue as potential angiogenic cell sources for bladder angiogenesis. PLoS ONE.

[6] Takizawa S, Nagata E, Nakayama T, Masuda H, Asahara T (2016). Recent progress in endothelial progenitor cell culture systems: potential for stroke therapy. Neurol Med Chir.

[7] Patschan D, Kribben A, Muller GA (2016). Postischemic microvasculopathy and endothelial progenitor cell-based therapy in ischemic AKI: update and perspectives. Am J Physiol Renal Physiol.

[8] Yang JX, Pan YY, Zhao YY, Wang XX (2013). Endothelial progenitor cell-based therapy for pulmonary arterial hypertension. Cell Transplant.

[9] Patel NA, Moss LD, Lee JY, Tajiri N, Acosta S, Hudson C, Parag S, Cooper DR, Borlongan CV, Bickford PC (2018). Long noncoding RNA MALAT1 in exosomes drives regenerative function and modulates inflammation-linked networks following traumatic brain injury. J Neuroinflammation.

[10] Pan BT, Johnstone RM (1983). Fate of the transferrin receptor during maturation of sheep reticulocytes in vitro: selective externalization of the receptor. Cell.

[11] Valadi H, Ekstrom K, Bossios A, Sjostrand M, Lee JJ, Lotvall JO (2007). Exosome-mediated transfer of mRNAs and microRNAs is a novel mechanism of genetic exchange between cells. Nat Cell Biol.

[12] Heilmann C, Beyersdorf F, Lutter G (2002). Collateral growth: cells arrive at the construction site. Cardiovasc Surg.

[13] Herberts CA, Kwa MS, Hermsen HP (2011). Risk factors in the development of stem cell therapy. J Transl Med.

[14] Yong T, Zhang X, Bie N, Zhang H, Zhang X, Li F, Hakeem A, Hu J, Gan L, Santos HA, Yang X (2019). Tumor exosome-based nanoparticles are efficient drug carriers for chemotherapy. Nat Commun.

[15] Cunnane EM, Weinbaum JS, O'Brien FJ, Vorp DA (2018). Future perspectives on the role of stem cells and extracellular vesicles in vascular tissue regeneration. Front Cardiovasc Med.

[16] Todorova D, Simoncini S, Lacroix R, Sabatier F, Dignat-George F (2017). Extracellular vesicles in angiogenesis. Circ Res.

[17] Fleury A, Martinez MC, Le Lay S (2014). Extracellular vesicles as therapeutic tools in cardiovascular diseases. Front Immunol.

[18] Fadini GP (2014). A reappraisal of the role of circulating (progenitor) cells in the pathobiology of diabetic complications. Diabetologia.

[19] Fadini GP, Losordo D, Dimmeler S (2012). Critical reevaluation of endothelial progenitor cell phenotypes for therapeutic and diagnostic use. Circ Res.

[20] Kim SW, Jin HL, Kang SM, Kim S, Yoo KJ, Jang Y, Kim HO, Yoon YS (2016). Therapeutic effects of late outgrowth endothelial progenitor cells or mesenchymal stem cells derived from human umbilical cord blood on infarct repair. Int J Cardiol.

[21] Pysna A, Bem R, Nemcova A, Fejfarova V, Jirkovska A, Hazdrova J, Jude EB, Dubsky M (2019). Endothelial progenitor cells biology in diabetes mellitus and peripheral arterial disease and their therapeutic potential. Stem Cell Rev Rep.

[22] Hristov M, Weber C (2004). Endothelial progenitor cells: characterization, pathophysiology, and possible clinical relevance. J Cell Mol Med.

[23] Aicher A, Zeiher AM, Dimmeler S (2005). Mobilizing endothelial progenitor cells. Hypertension.

[24] de la Puente P, Muz B, Azab F, Azab AK (2013). Cell trafficking of endothelial progenitor cells in tumor progression. Clin Cancer Res.

[25] Raposo G, Stoorvogel W (2013). Extracellular vesicles: exosomes, microvesicles, and friends. J Cell Biol.

[26] Cocucci E, Meldolesi J (2015). Ectosomes and exosomes: shedding the confusion between extracellular vesicles. Trends Cell Biol.

[27] Xing C, Yin P, Peng Z, Zhang H (2020). Engineering mono-chalcogen nanomaterials for omnipotent anticancer applications: progress and challenges. Adv Healthc Mater.

[28] McAndrews KM, Kalluri R (2019). Mechanisms associated with biogenesis of exosomes in cancer. Mol Cancer.

[29] Kharaziha P, Ceder S, Li Q, Panaretakis T (2012). Tumor cell-derived exosomes: a message in a bottle. Biochim Biophys Acta.

[30] Mathieu M, Martin-Jaular L, Lavieu G, Thery C (2019). Specificities of secretion and uptake of exosomes and other extracellular vesicles for cell-to-cell communication. Nat Cell Biol.

[31] van der Pol E, Boing AN, Harrison P, Sturk A, Nieuwland R (2012). Classification, functions, and clinical relevance of extracellular vesicles. Pharmacol Rev.

[32] Muralidharan-Chari V, Clancy JW, Sedgwick A, D'Souza-Schorey C (2010). Microvesicles: mediators of extracellular communication during cancer progression. J Cell Sci.

[33] Beyer C, Pisetsky DS (2010). The role of microparticles in the pathogenesis of rheumatic diseases. Nat Rev Rheumatol.

[34] Sebbagh M, Renvoize C, Hamelin J, Riche N, Bertoglio J, Breard J (2001). Caspase-3-mediated cleavage of ROCK I induces MLC phosphorylation and apoptotic membrane blebbing. Nat Cell Biol.

[35] Coleman ML, Sahai EA, Yeo M, Bosch M, Dewar A, Olson MF (2001). Membrane blebbing during apoptosis results from caspase-mediated activation of ROCK I. Nat Cell Biol.

[36] Kang YT, Purcell E, Palacios-Rolston C, Lo TW, Ramnath N, Jolly S, Nagrath S (2019). Isolation and profiling of circulating tumor-associated exosomes using extracellular vesicular lipid-protein binding affinity based microfluidic device. Small.

[37] Chen BY, Sung CW, Chen C, Cheng CM, Lin DP, Huang CT, Hsu MY (2019). Advances in exosomes technology. Clin Chim Acta.

[38] Nath Neerukonda S, Egan NA, Patria J, Assakhi I, Tavlarides-Hontz P, Modla S, Munoz ER, Hudson MB, Parcells MS (2019). Comparison of exosomes purified via ultracentrifugation (UC) and total exosome isolation (TEI) reagent from the serum of Marek's disease virus (MDV)-vaccinated and tumor-bearing chickens. J Virol Methods.

[39] Garcia-Romero N, Madurga R, Rackov G, Palacin-Aliana I, Nunez-Torres R, Asensi-Puig A, Carrion-Navarro J, Esteban-Rubio S, Peinado H, Gonzalez-Neira A (2019). Polyethylene glycol improves current methods for circulating extracellular vesicle-derived DNA isolation. J Transl Med.

[40] Patel GK, Khan MA, Zubair H, Srivastava SK, Khushman M, Singh S, Singh AP (2019). Comparative analysis of exosome isolation methods using culture supernatant for optimum yield, purity and downstream applications. Sci Rep.

[41] Vergauwen G, Dhondt B, Van Deun J, De Smedt E, Berx G, Timmerman E, Gevaert K, Miinalainen I, Cocquyt V, Braems G (2017). Confounding factors of ultrafiltration and protein analysis in extracellular vesicle research. Sci Rep.

[42] Foers AD, Chatfield S, Dagley LF, Scicluna BJ, Webb AI, Cheng L, Hill AF, Wicks IP, Pang KC (2018). Enrichment of extracellular vesicles from human synovial fluid using size exclusion chromatography. J Extracell Vesicles.

[43] Gamez-Valero A, Monguio-Tortajada M, Carreras-Planella L, Franquesa M, Beyer K, Borras FE (2016). Size-exclusion chromatography-based isolation minimally alters extracellular vesicles' characteristics compared to precipitating agents. Sci Rep.

[44] Salih M, Zietse R, Hoorn EJ (2014). Urinary extracellular vesicles and the kidney: biomarkers and beyond. Am J Physiol Renal Physiol.

[45] Konoshenko MY, Lekchnov EA, Vlassov AV, Laktionov PP (2018). Isolation of extracellular vesicles: general methodologies and latest trends. Biomed Res Int.

[46] Doyle LM, Wang MZ (2019). Overview of extracellular vesicles, their origin, composition, purpose, and methods for exosome isolation and analysis. Cells.

[47] Xing Z, Zhao C, Liu H, Fan Y (2020). Endothelial progenitor cell-derived extracellular vesicles: a novel candidate for regenerative medicine and disease treatment. Adv Healthc Mater.

[48] Zhang C, Guo F, Chang M, Zhou Z, Yi L, Gao C, Huang X, Huan J (2019). Exosome-delivered syndecan-1 rescues acute lung injury via a FAK/p190RhoGAP/RhoA/ROCK/NF-kappaB signaling axis and glycocalyx enhancement. Exp Cell Res.

[49] Wang Y, Zhang L, Li Y, Chen L, Wang X, Guo W, Zhang X, Qin G, He SH, Zimmerman A (2015). Exosomes/microvesicles from induced pluripotent stem cells deliver cardioprotective miRNAs and prevent cardiomyocyte apoptosis in the ischemic myocardium. Int J Cardiol.

[50] Rayamajhi S, Nguyen TDT, Marasini R, Aryal S (2019). Macrophage-derived exosome-mimetic hybrid vesicles for tumor targeted drug delivery. Acta Biomater.

[51] Harshman SW, Canella A, Ciarlariello PD, Agarwal K, Branson OE, Rocci A, Cordero H, Phelps MA, Hade EM, Dubovsky JA (2016). Proteomic characterization of circulating extracellular vesicles identifies novel serum myeloma associated markers. J Proteomics.

[52] Pisitkun T, Shen RF, Knepper MA (2004). Identification and proteomic profiling of exosomes in human urine. Proc Natl Acad Sci U S A.

[53] Bian X, Xiao YT, Wu T, Yao M, Du L, Ren S, Wang J (2019). Microvesicles and chemokines in tumor microenvironment: mediators of intercellular communications in tumor progression. Mol Cancer.

[54] Kosaka N, Izumi H, Sekine K, Ochiya T (2010). microRNA as a new immune-regulatory agent in breast milk. Silence.

[55] Palanisamy V, Sharma S, Deshpande A, Zhou H, Gimzewski J, Wong DT (2010). Nanostructural and transcriptomic analyses of human saliva derived exosomes. PLoS ONE.

[56] Schaaij-Visser TB, de Wit M, Lam SW, Jimenez CR (2013). The cancer secretome, current status and opportunities in the lung, breast and colorectal cancer context. Biochim Biophys Acta.

[57] Logozzi M, De Milito A, Lugini L, Borghi M, Calabro L, Spada M, Perdicchio M, Marino ML, Federici C, Iessi E (2009). High levels of exosomes expressing CD63 and caveolin-1 in plasma of melanoma patients. PLoS ONE.

[58] Shao H, Chung J, Balaj L, Charest A, Bigner DD, Carter BS, Hochberg FH, Breakefield XO, Weissleder R, Lee H (2012). Protein typing of circulating microvesicles allows real-time monitoring of glioblastoma therapy. Nat Med.

[59] Im H, Shao H, Park YI, Peterson VM, Castro CM, Weissleder R, Lee H (2014). Label-free detection and molecular profiling of exosomes with a nano-plasmonic sensor. Nat Biotechnol.

[60] Ogata-Kawata H, Izumiya M, Kurioka D, Honma Y, Yamada Y, Furuta K, Gunji T, Ohta H, Okamoto H, Sonoda H (2014). Circulating exosomal microRNAs as biomarkers of colon cancer. PLoS ONE.

[61] Matsumura T, Sugimachi K, Iinuma H, Takahashi Y, Kurashige J, Sawada G, Ueda M, Uchi R, Ueo H, Takano Y (2015). Exosomal microRNA in serum is a novel biomarker of recurrence in human colorectal cancer. Br J Cancer.

[62] Raposo G, Nijman HW, Stoorvogel W, Liejendekker R, Harding CV, Melief CJ, Geuze HJ (1996). B lymphocytes secrete antigen-presenting vesicles. J Exp Med.

[63] Samuelson I, Vidal-Puig AJ (2018). Fed-EXosome: extracellular vesicles and cell-cell communication in metabolic regulation. Essays Biochem.

[64] Denham J, Spencer SJ (2020). Emerging roles of extracellular vesicles in the intercellular communication for exercise-induced adaptations. Am J Physiol Endocrinol Metab.

[65] Capra E, Lange-Consiglio A (2020). The biological function of extracellular vesicles during fertilization, early embryo-maternal crosstalk and their involvement in reproduction: review and overview. Biomolecules.

[66] Bebelman MP, Smit MJ, Pegtel DM, Baglio SR (2018). Biogenesis and function of extracellular vesicles in cancer. Pharmacol Ther.

[67] Quail DF, Joyce JA (2013). Microenvironmental regulation of tumor progression and metastasis. Nat Med.

[68] Guo W, Gao Y, Li N, Shao F, Wang C, Wang P, Yang Z, Li R, He J (2017). Exosomes: new players in cancer (Review). Oncol Rep.

[69] Baglio SR, Lagerweij T, Perez-Lanzon M, Ho XD, Leveille N, Melo SA, Cleton-Jansen AM, Jordanova ES, Roncuzzi L, Greco M (2017). Blocking tumor-educated MSC paracrine activity halts osteosarcoma progression. Clin Cancer Res.

[70] Lugini L, Valtieri M, Federici C, Cecchetti S, Meschini S, Condello M, Signore M, Fais S (2016). Exosomes from human colorectal cancer induce a tumor-like behavior in colonic mesenchymal stromal cells. Oncotarget.

[71] Webber JP, Spary LK, Sanders AJ, Chowdhury R, Jiang WG, Steadman R, Wymant J, Jones AT, Kynaston H, Mason MD (2015). Differentiation of tumour-promoting stromal myofibroblasts by cancer exosomes. Oncogene.

[72] Cho JA, Park H, Lim EH, Lee KW (2012). Exosomes from breast cancer cells can convert adipose tissue-derived mesenchymal stem cells into myofibroblast-like cells. Int J Oncol.

[73] Richards KE, Zeleniak AE, Fishel ML, Wu J, Littlepage LE, Hill R (2017). Cancer-associated fibroblast exosomes regulate survival and proliferation of pancreatic cancer cells. Oncogene.

[74] Al-Nedawi K, Meehan B, Micallef J, Lhotak V, May L, Guha A, Rak J (2008). Intercellular transfer of the oncogenic receptor EGFRvIII by microvesicles derived from tumour cells. Nat Cell Biol.

[75] Kosaka N, Yoshioka Y, Fujita Y, Ochiya T (2016). Versatile roles of extracellular vesicles in cancer. J Clin Invest.

[76] Ciravolo V, Huber V, Ghedini GC, Venturelli E, Bianchi F, Campiglio M, Morelli D, Villa A, Della Mina P, Menard S (2012). Potential role of HER2-overexpressing exosomes in countering trastuzumab-based therapy. J Cell Physiol.

[77] Marleau AM, Chen CS, Joyce JA, Tullis RH (2012). Exosome removal as a therapeutic adjuvant in cancer. J Transl Med.

[78] Chen G, Huang AC, Zhang W, Zhang G, Wu M, Xu W, Yu Z, Yang J, Wang B, Sun H (2018). Exosomal PD-L1 contributes to immunosuppression and is associated with anti-PD-1 response. Nature.

[79] Middleton RC, Rogers RG, De Couto G, Tseliou E, Luther K, Holewinski R, Soetkamp D, Van Eyk JE, Antes TJ, Marban E (2018). Newt cells secrete extracellular vesicles with therapeutic bioactivity in mammalian cardiomyocytes. J Extracell Vesicles.

[80] Escrevente C, Keller S, Altevogt P, Costa J (2011). Interaction and uptake of exosomes by ovarian cancer cells. BMC Cancer.

[81] Terriaca S, Fiorelli E, Scioli MG, Fabbri G, Storti G, Cervelli V, Orlandi A (2021). Endothelial progenitor cell-derived extracellular vesicles: potential therapeutic application in tissue repair and regeneration. Int J Mol Sci.

[82] Kowal J, Arras G, Colombo M, Jouve M, Morath JP, Primdal-Bengtson B, Dingli F, Loew D, Tkach M, Thery C (2016). Proteomic comparison defines novel markers to characterize heterogeneous populations of extracellular vesicle subtypes. Proc Natl Acad Sci U S A.

[83] Hoste EAJ, Kellum JA, Katz NM, Rosner MH, Haase M, Ronco C (2010). Epidemiology of acute kidney injury. Contrib Nephrol.

[84] Liu J, Dou Q, Zhou C, Zhou L, Zhao F, Xu L, Xu Z, Ge Y, Wu R, Jia R (2020). Low-energy shock wave pretreatment recruit circulating endothelial progenitor cells to attenuate renal ischaemia reperfusion injury. J Cell Mol Med.

[85] Zhu Y, Zhao K, Wang L, Lu T, Zhou C, Ge Y, Wu R, Jia R, Zheng C (2020). Erythropoietin preconditioning mobilizes endothelial progenitor cells to attenuate nephron-sparing surgery-induced ischemia-reperfusion injury. Transplant Proc.

[86] Xue J, Qin Z, Li X, Cao P, Jia R (2017). Protective effects of ischemic preconditioning-mediated homing of endothelial progenitor cells on renal acute ischemia and reperfusion injury in male Rats. Ann Transplant.

[87] Chen B, Bo CJ, Jia RP, Liu H, Wu R, Wu J, Ge YZ, Teng GJ (2013). The renoprotective effect of bone marrow-derived endothelial progenitor cell transplantation on acute ischemia-reperfusion injury in rats. Transplant Proc.

[88] Bo CJ, Chen B, Jia RP, Zhu JG, Cao P, Liu H, Wu R, Ge YZ, Wu JP (2013). Effects of ischemic preconditioning in the late phase on homing of endothelial progenitor cells in renal ischemia/reperfusion injury. Transplant Proc.

[89] Liu H, Wu R, Jia RP, Zhong B, Zhu JG, Yu P, Zhao Y, Ge YZ, Wu JP (2013). Ischemic preconditioning increases endothelial progenitor cell number to attenuate partial nephrectomy-induced ischemia/reperfusion injury. PLoS ONE.

[90] Li B, Cohen A, Hudson TE, Motlagh D, Amrani DL, Duffield JS (2010). Mobilized human hematopoietic stem/progenitor cells promote kidney repair after ischemia/reperfusion injury. Circulation.

[91] Cantaluppi V, Gatti S, Medica D, Figliolini F, Bruno S, Deregibus MC, Sordi A, Biancone L, Tetta C, Camussi G (2012). Microvesicles derived from endothelial progenitor cells protect the kidney from ischemia-reperfusion injury by microRNA-dependent reprogramming of resident renal cells. Kidney Int.

[92] Zarjou A, Agarwal A (2011). Sepsis and acute kidney injury. J Am Soc Nephrol.

[93] Venet F, Monneret G (2018). Advances in the understanding and treatment of sepsis-induced immunosuppression. Nat Rev Nephrol.

[94] He Z, Wang H, Yue L (2020). Endothelial progenitor cells-secreted extracellular vesicles containing microRNA-93-5p confer protection against sepsis-induced acute kidney injury via the KDM6B/H3K27me3/TNF-alpha axis. Exp Cell Res.

[95] Zhang Y, Huang H, Liu W, Liu S, Wang XY, Diao ZL, Zhang AH, Guo W, Han X, Dong X, Katilov O (2021). Endothelial progenitor cells-derived exosomal microRNA-21-5p alleviates sepsis-induced acute kidney injury by inhibiting RUNX1 expression. Cell Death Dis.

[96] Chadban SJ, Atkins RC (2005). Glomerulonephritis. Lancet.

[97] Cantaluppi V, Medica D, Mannari C, Stiaccini G, Figliolini F, Dellepiane S, Quercia AD, Migliori M, Panichi V, Giovannini L (2015). Endothelial progenitor cell-derived extracellular vesicles protect from complement-mediated mesangial injury in experimental anti-Thy1.1 glomerulonephritis. Nephrol Dial Transplant.

[98] Medica D, Franzin R, Stasi A, Castellano G, Migliori M, Panichi V, Figliolini F, Gesualdo L, Camussi G, Cantaluppi V (2021). Extracellular vesicles derived from endothelial progenitor cells protect human glomerular endothelial cells and podocytes from complement- and cytokine-mediated injury. Cells.

[99] Yang J, Wang M, Zhu F, Sun J, Xu H, Shin CL, Shin OL, Zhao Z, Pei G, Zhu H, Cao C (2019). Putative endothelial progenitor cells do not promote vascular repair but attenuate pericyte-myofibroblast transition in UUO-induced renal fibrosis. Stem Cell Res Ther.

[100] Perkins GD, Nathani N, Richter AG, Park D, Shyamsundar M, Heljasvaara R, Pihlajaniemi T, Manji M, Tunnicliffe W, McAuley D (2009). Type XVIII collagen degradation products in acute lung injury. Crit Care.

[101] Hristov M, Erl W, Weber PC (2003). Endothelial progenitor cells: mobilization, differentiation, and homing. Arterioscler Thromb Vasc Biol.

[102] Wu X, Liu Z, Hu L, Gu W, Zhu L (2018). Exosomes derived from endothelial progenitor cells ameliorate acute lung injury by transferring miR-126. Exp Cell Res.

[103] Zhou Y, Li P, Goodwin AJ, Cook JA, Halushka PV, Chang E, Zingarelli B, Fan H (2019). Exosomes from endothelial progenitor cells improve outcomes of the lipopolysaccharide-induced acute lung injury. Crit Care.

[104] Mauffrey C, Barlow BT, Smith W (2015). Management of segmental bone defects. J Am Acad Orthop Surg.

[105] Spiegl U, Patzold R, Friederichs J, Hungerer S, Militz M, Buhren V (2013). Clinical course, complication rate and outcome of segmental resection and distraction osteogenesis after chronic tibial osteitis. Injury.

[106] Jia Y, Zhu Y, Qiu S, Xu J, Chai Y (2019). Exosomes secreted by endothelial progenitor cells accelerate bone regeneration during distraction osteogenesis by stimulating angiogenesis. Stem Cell Res Ther.

[107] Qin Y, Zhang C (2017). Endothelial progenitor cellderived extracellular vesiclemeditated celltocell communication regulates the proliferation and osteoblastic differentiation of bone mesenchymal stromal cells. Mol Med Rep.

[108] Cui Y, Fu S, Sun D, Xing J, Hou T, Wu X (2019). EPC-derived exosomes promote osteoclastogenesis through LncRNA-MALAT1. J Cell Mol Med.

[109] Buckley L, Guyatt G, Fink HA, Cannon M, Grossman J, Hansen KE, Humphrey MB, Lane NE, Magrey M, Miller M (2017). 2017 American college of rheumatology guideline for the prevention and treatment of glucocorticoid-induced osteoporosis. Arthritis Care Res.

[110] Lu J, Yang J, Zheng Y, Chen X, Fang S (2019). Extracellular vesicles from endothelial progenitor cells prevent steroid-induced osteoporosis by suppressing the ferroptotic pathway in mouse osteoblasts based on bioinformatics evidence. Sci Rep.

[111] Jiang J, Wang W, Sun YJ, Hu M, Li F, Zhu DY (2007). Neuroprotective effect of curcumin on focal cerebral ischemic rats by preventing blood-brain barrier damage. Eur J Pharmacol.

[112] Lakhan SE, Kirchgessner A, Hofer M (2009). Inflammatory mechanisms in ischemic stroke: therapeutic approaches. J Transl Med.

[113] Broughton BR, Reutens DC, Sobey CG (2009). Apoptotic mechanisms after cerebral ischemia. Stroke.

[114] Margaill I, Plotkine M, Lerouet D (2005). Antioxidant strategies in the treatment of stroke. Free Radic Biol Med.

[115] Wang J, Chen S, Ma X, Cheng C, Xiao X, Chen J, Liu S, Zhao B, Chen Y (2013). Effects of endothelial progenitor cell-derived microvesicles on hypoxia/reoxygenation-induced endothelial dysfunction and apoptosis. Oxid Med Cell Longev.

[116] Ma X, Wang J, Li J, Ma C, Chen S, Lei W, Yang Y, Liu S, Bihl J, Chen C (2018). Loading MiR-210 in endothelial progenitor cells derived exosomes boosts their beneficial effects on hypoxia/reoxygeneation-injured human endothelial cells via protecting mitochondrial function. Cell Physiol Biochem.

[117] Li Y, Wang J, Chen S, Wu P, Xu S, Wang C, Shi H, Bihl J (2020). miR-137 boosts the neuroprotective effect of endothelial progenitor cell-derived exosomes in oxyhemoglobin-treated SH-SY5Y cells partially via COX2/PGE2 pathway. Stem Cell Res Ther.

[118] Garbuzova-Davis S, Kurien C, Thomson A, Falco D, Ahmad S, Staffetti J, Steiner G, Abraham S, James G, Mahendrasah A (2017). Endothelial and astrocytic support by human bone marrow stem cell grafts into symptomatic ALS mice towards blood-spinal cord barrier repair. Sci Rep.

[119] Garbuzova-Davis S, Ehrhart J, Mustafa H, Llauget A, Boccio KJ, Sanberg PR, Appel SH, Borlongan CV (2019). Phenotypic characteristics of human bone marrow-derived endothelial progenitor cells in vitro support cell effectiveness for repair of the blood-spinal cord barrier in ALS. Brain Res.

[120] Garbuzova-Davis S, Kurien C, Haller E, Eve DJ, Navarro S, Steiner G, Mahendrasah A, Hailu S, Khatib M, Boccio KJ (2019). Human bone marrow endothelial progenitor cell transplantation into symptomatic ALS mice delays disease progression and increases motor neuron survival by repairing blood-spinal cord barrier. Sci Rep.

[121] Garbuzova-Davis S, Willing AE, Ehrhart J, Wang L, Sanberg PR, Borlongan CV (2020). Cell-free extracellular vesicles derived from human bone marrow endothelial progenitor cells as potential therapeutics for microvascular endothelium restoration in ALS. Neuromolecular Med.

[122] Zou J, Fei Q, Xiao H, Wang H, Liu K, Liu M, Zhang H, Xiao X, Wang K, Wang N (2019). VEGF-A promotes angiogenesis after acute myocardial infarction through increasing ROS production and enhancing ER stress-mediated autophagy. J Cell Physiol.

[123] Furtado MB, Costa MW, Rosenthal NA (2016). The cardiac fibroblast: origin, identity and role in homeostasis and disease. Differentiation.

[124] Ke X, Yang D, Liang J, Wang X, Wu S, Wang X, Hu C (2017). Human endothelial progenitor cell-derived exosomes increase proliferation and angiogenesis in cardiac fibroblasts by promoting the mesenchymal-endothelial transition and reducing high mobility group box 1 protein B1 expression. DNA Cell Biol.

[125] Ke X, Yang R, Wu F, Wang X, Liang J, Hu X, Hu C (2021). Exosomal miR-218-5p/miR-363-3p from endothelial progenitor cells ameliorate myocardial infarction by targeting the p53/JMY signaling pathway. Oxid Med Cell Longev.

[126] Huang Y, Chen L, Feng Z, Chen W, Yan S, Yang R, Xiao J, Gao J, Zhang D, Ke X (2021). EPC-derived exosomal miR-1246 and miR-1290 regulate phenotypic changes of fibroblasts to endothelial cells to exert protective effects on myocardial infarction by targeting ELF5 and SP1. Front Cell Dev Biol.

[127] Yue Y, Wang C, Benedict C, Huang G, Truongcao M, Roy R, Cimini M, Garikipati VNS, Cheng Z, Koch WJ, Kishore R (2020). Interleukin-10 deficiency alters endothelial progenitor cell-derived exosome reparative effect on myocardial repair via integrin-linked kinase enrichment. Circ Res.

[128] Liu Y, Xu J, Gu R, Li Z, Wang K, Qi Y, Sun X, Xie J, Wang L, Xu B, Kang L (2020). Circulating exosomal miR-144-3p inhibits the mobilization of endothelial progenitor cells post myocardial infarction via regulating the MMP9 pathway. Aging.

[129] Sahoo S, Klychko E, Thorne T, Misener S, Schultz KM, Millay M, Ito A, Liu T, Kamide C, Agrawal H (2011). Exosomes from human CD34(+) stem cells mediate their proangiogenic paracrine activity. Circ Res.

[130] Gnecchi M, He H, Liang OD, Melo LG, Morello F, Mu H, Noiseux N, Zhang L, Pratt RE, Ingwall JS, Dzau VJ (2005). Paracrine action accounts for marked protection of ischemic heart by Akt-modified mesenchymal stem cells. Nat Med.

[131] Chen CW, Wang LL, Zaman S, Gordon J, Arisi MF, Venkataraman CM, Chung JJ, Hung G, Gaffey AC, Spruce LA (2018). Sustained release of endothelial progenitor cell-derived extracellular vesicles from shear-thinning hydrogels improves angiogenesis and promotes function after myocardial infarction. Cardiovasc Res.

[132] Chung JJ, Han J, Wang LL, Arisi MF, Zaman S, Gordon J, Li E, Kim ST, Tran Z, Chen CW (2020). Delayed delivery of endothelial progenitor cell-derived extracellular vesicles via shear thinning gel improves postinfarct hemodynamics. J Thorac Cardiovasc Surg.

[133] Jansen F, Yang X, Hoelscher M, Cattelan A, Schmitz T, Proebsting S, Wenzel D, Vosen S, Franklin BS, Fleischmann BK (2013). Endothelial microparticle-mediated transfer of MicroRNA-126 promotes vascular endothelial cell repair via SPRED1 and is abrogated in glucose-damaged endothelial microparticles. Circulation.

[134] Li X, Chen C, Wei L, Li Q, Niu X, Xu Y, Wang Y, Zhao J (2016). Exosomes derived from endothelial progenitor cells attenuate vascular repair and accelerate reendothelialization by enhancing endothelial function. Cytotherapy.

[135] Xu BY, Xiang MX, Wang JA (2015). Endothelial progenitor cells and in-stent restenosis. Curr Stem Cell Res Ther.

[136] Kong J, Wang F, Zhang J, Cui Y, Pan L, Zhang W, Wen J, Liu P (2018). Exosomes of endothelial progenitor cells inhibit neointima formation after carotid artery injury. J Surg Res.

[137] Hu H, Jiang C, Li R, Zhao J (2019). Comparison of endothelial cell- and endothelial progenitor cell-derived exosomes in promoting vascular endothelial cell repair. Int J Clin Exp Pathol.

[138] Hu H, Wang B, Jiang C, Li R, Zhao J (2019). Endothelial progenitor cell-derived exosomes facilitate vascular endothelial cell repair through shuttling miR-21-5p to modulate thrombospondin-1 expression. Clin Sci.

[139] Yang SW, Hennessy RR, Khosla S, Lennon R, Loeffler D, Sun T, Liu Z, Park KH, Wang FL, Lerman LO, Lerman A (2017). Circulating osteogenic endothelial progenitor cell counts: new biomarker for the severity of coronary artery disease. Int J Cardiol.

[140] Yi M, Wu Y, Long J, Liu F, Liu Z, Zhang YH, Sun XP, Fan ZX, Gao J, Si J (2019). Exosomes secreted from osteocalcin-overexpressing endothelial progenitor cells promote endothelial cell angiogenesis. Am J Physiol Cell Physiol.

[141] Hill MF, Singal PK (1996). Antioxidant and oxidative stress changes during heart failure subsequent to myocardial infarction in rats. Am J Pathol.

[142] Wang J, Chen S, Bihl J (2020). Exosome-mediated transfer of ACE2 (angiotensin-converting enzyme 2) from endothelial progenitor cells promotes survival and function of endothelial cell. Oxid Med Cell Longev.

[143] Wang J, Li J, Cheng C, Liu S (2020). Angiotensin-converting enzyme 2 augments the effects of endothelial progenitor cells-exosomes on vascular smooth muscle cell phenotype transition. Cell Tissue Res.

[144] Jobe AH, Bancalari E (2001). Bronchopulmonary dysplasia. Am J Respir Crit Care Med.

[145] Zhang X, Lu A, Li Z, Sun J, Dai D, Qian L (2019). Exosomes secreted by endothelial progenitor cells improve the bioactivity of pulmonary microvascular endothelial cells exposed to hyperoxia in vitro. Ann Transl Med.

[146] Li L, Wang H, Zhang J, Chen X, Zhang Z, Li Q (2021). Effect of endothelial progenitor cell-derived extracellular vesicles on endothelial cell ferroptosis and atherosclerotic vascular endothelial injury. Cell Death Discov.

[147] Fink MP, Warren HS (2014). Strategies to improve drug development for sepsis. Nat Rev Drug Discov.

[148] Fan H, Goodwin AJ, Chang E, Zingarelli B, Borg K, Guan S, Halushka PV, Cook JA (2014). Endothelial progenitor cells and a stromal cell-derived factor-1alpha analogue synergistically improve survival in sepsis. Am J Respir Crit Care Med.

[149] Zhou Y, Li P, Goodwin AJ, Cook JA, Halushka PV, Chang E, Fan H (2018). Exosomes from endothelial progenitor cells improve the outcome of a murine model of sepsis. Mol Ther.

[150] Hong X, Wang J, Li S, Zhao Z, Feng Z (2021). MicroRNA-375-3p in endothelial progenitor cells-derived extracellular vesicles relieves myocardial injury in septic rats via BRD4-mediated PI3K/AKT signaling pathway. Int Immunopharmacol.

[151] Cheng L, Nan C, Kang L, Zhang N, Liu S, Chen H, Hong C, Chen Y, Liang Z, Liu X (2020). Whole blood transcriptomic investigation identifies long non-coding RNAs as regulators in sepsis. J Transl Med.

[152] Qiu N, Xu X, He Y (2020). LncRNA TUG1 alleviates sepsis-induced acute lung injury by targeting miR-34b-5p/GAB1. BMC Pulm Med.

[153] Ma W, Zhang W, Cui B, Gao J, Liu Q, Yao M, Ning H, Xing L (2021). Functional delivery of lncRNA TUG1 by endothelial progenitor cells derived extracellular vesicles confers anti-inflammatory macrophage polarization in sepsis via impairing miR-9-5p-targeted SIRT1 inhibition. Cell Death Dis.

[154] Kharroubi AT, Darwish HM (2015). Diabetes mellitus: the epidemic of the century. World J Diabetes.

[155] Patel S, Srivastava S, Singh MR, Singh D (2019). Mechanistic insight into diabetic wounds: pathogenesis, molecular targets and treatment strategies to pace wound healing. Biomed Pharmacother.

[156] Lioupis C (2005). Effects of diabetes mellitus on wound healing: an update. J Wound Care.

[157] Li X, Jiang C, Zhao J (2016). Human endothelial progenitor cells-derived exosomes accelerate cutaneous wound healing in diabetic rats by promoting endothelial function. J Diabetes Complicat.

[158] Zhang J, Chen C, Hu B, Niu X, Liu X, Zhang G, Zhang C, Li Q, Wang Y (2016). Exosomes derived from human endothelial progenitor cells accelerate cutaneous wound healing by promoting angiogenesis through Erk1/2 signaling. Int J Biol Sci.

[159] Xu J, Bai S, Cao Y, Liu L, Fang Y, Du J, Luo L, Chen M, Shen B, Zhang Q (2020). miRNA-221-3p in endothelial progenitor cell-derived exosomes accelerates skin wound healing in diabetic mice. Diabetes Metab Syndr Obes.

[160] Kang DH, Yoon W (2019). Current opinion on endovascular therapy for emergent large vessel occlusion due to underlying intracranial atherosclerotic stenosis. Korean J Radiol.

[161] Wu K, Yang Y, Zhong Y, Ammar HM, Zhang P, Guo R, Liu H, Cheng C, Koroscil TM, Chen Y (2016). The effects of microvesicles on endothelial progenitor cells are compromised in type 2 diabetic patients via downregulation of the miR-126/VEGFR2 pathway. Am J Physiol Endocrinol Metab.

[162] Wang J, Chen S, Zhang W, Chen Y, Bihl JC (2020). Exosomes from miRNA-126-modified endothelial progenitor cells alleviate brain injury and promote functional recovery after stroke. CNS Neurosci Ther.

[163] Tang N, Jiang S, Yang Y, Liu S, Ponnusamy M, Xin H, Yu T (2018). Noncoding RNAs as therapeutic targets in atherosclerosis with diabetes mellitus. Cardiovasc Ther.

[164] Bai S, Yin Q, Dong T, Dai F, Qin Y, Ye L, Du J, Zhang Q, Chen H, Shen B (2020). Endothelial progenitor cell-derived exosomes ameliorate endothelial dysfunction in a mouse model of diabetes. Biomed Pharmacother.

[165] Fu X, Liu G, Halim A, Ju Y, Luo Q, Song AG (2019). Mesenchymal stem cell migration and tissue repair. Cells.

[166] Wong SP, Rowley JE, Redpath AN, Tilman JD, Fellous TG, Johnson JR (2015). Pericytes, mesenchymal stem cells and their contributions to tissue repair. Pharmacol Ther.

[167] Rana D, Kumar A, Sharma S (2018). Endothelial progenitor cells as molecular targets in vascular senescence and repair. Curr Stem Cell Res Ther.

[168] Ong SG, Wu JC (2015). Exosomes as potential alternatives to stem cell therapy in mediating cardiac regeneration. Circ Res.

[169] Salybekov AA, Kunikeyev AD, Kobayashi S, Asahara T (2021). Latest advances in endothelial progenitor cell-derived extracellular vesicles translation to the clinic. Front Cardiovasc Med.

[170] Tian Y, Li S, Song J, Ji T, Zhu M, Anderson GJ, Wei J, Nie G (2014). A doxorubicin delivery platform using engineered natural membrane vesicle exosomes for targeted tumor therapy. Biomaterials.

[171] Zitvogel L, Regnault A, Lozier A, Wolfers J, Flament C, Tenza D, Ricciardi-Castagnoli P, Raposo G, Amigorena S (1998). Eradication of established murine tumors using a novel cell-free vaccine: dendritic cell-derived exosomes. Nat Med.

[172] Riazifar M, Mohammadi MR, Pone EJ, Yeri A, Lasser C, Segaliny AI, McIntyre LL, Shelke GV, Hutchins E, Hamamoto A (2019). Stem cell-derived exosomes as nanotherapeutics for autoimmune and neurodegenerative disorders. ACS Nano.

[173] Zhu LP, Tian T, Wang JY, He JN, Chen T, Pan M, Xu L, Zhang HX, Qiu XT, Li CC (2018). Hypoxia-elicited mesenchymal stem cell-derived exosomes facilitates cardiac repair through miR-125b-mediated prevention of cell death in myocardial infarction. Theranostics.

[174] Huang B, Lu J, Ding C, Zou Q, Wang W, Li H (2018). Exosomes derived from human adipose mesenchymal stem cells improve ovary function of premature ovarian insufficiency by targeting SMAD. Stem Cell Res Ther.

[175] Vonk LA, van Dooremalen SFJ, Liv N, Klumperman J, Coffer PJ, Saris DBF, Lorenowicz MJ (2018). Mesenchymal stromal/stem cell-derived extracellular vesicles promote human cartilage regeneration in vitro. Theranostics.

[176] Miyasaki DM, Senegaglia AC, de Moura SAB, Leitolis A, Capriglione LGA, Fracaro L, Boldrini Leite LM, Utumi PH, Fragoso FYI, Meyer F (2022). Treatment of chronic kidney disease with extracellular vesicles from mesenchymal stem cells and CD133(+) expanded cells: a comparative preclinical analysis. Int J Mol Sci.

[177] Song Y, Bai Z, Zhang Y, Chen J, Chen M, Zhang Y, Zhang X, Mai H, Wang B, Lin Y, Gu S (2022). Protective effects of endothelial progenitor cell microvesicles on Ang IIinduced rat kidney cell injury. Mol Med Rep.

[178] Chopra H, Hung MK, Kwong DL, Zhang CF, Pow EHN (2018). Insights into endothelial progenitor cells: origin, classification, potentials, and prospects. Stem Cells Int.

[179] Cheng CC, Lo HH, Huang TS, Cheng YC, Chang ST, Chang SJ, Wang HW (2012). Genetic module and miRNome trait analyses reflect the distinct biological features of endothelial progenitor cells from different anatomic locations. BMC Genomics.

[180] Madlambayan G, Rogers I (2006). Umbilical cord-derived stem cells for tissue therapy: current and future uses. Regen Med.

[181] Deregibus MC, Cantaluppi V, Calogero R, Lo Iacono M, Tetta C, Biancone L, Bruno S, Bussolati B, Camussi G (2007). Endothelial progenitor cell derived microvesicles activate an angiogenic program in endothelial cells by a horizontal transfer of mRNA. Blood.

[182] Shin KO, Ha DH, Kim JO, Crumrine DA, Meyer JM, Wakefield JS, Lee Y, Kim B, Kim S, Kim HK (2020). Exosomes from human adipose tissue-derived mesenchymal stem cells promote epidermal barrier repair by inducing de novo synthesis of ceramides in atopic dermatitis. Cells.

[183] Li S, Liu J, Liu S, Jiao W, Wang X (2021). Mesenchymal stem cell-derived extracellular vesicles prevent the development of osteoarthritis via the circHIPK3/miR-124-3p/MYH9 axis. J Nanobiotechnology.

[184] Zhao M, Liu S, Wang C, Wang Y, Wan M, Liu F, Gong M, Yuan Y, Chen Y, Cheng J (2021). Mesenchymal stem cell-derived extracellular vesicles attenuate mitochondrial damage and inflammation by stabilizing mitochondrial DNA. ACS Nano.

[185] Adlerz K, Patel D, Rowley J, Ng K, Ahsan T (2020). Strategies for scalable manufacturing and translation of MSC-derived extracellular vesicles. Stem Cell Res.

[186] Paganini C, Capasso Palmiero U, Pocsfalvi G, Touzet N, Bongiovanni A, Arosio P (2019). Scalable production and isolation of extracellular vesicles: available sources and lessons from current industrial bioprocesses. Biotechnol J.

[187] Kooijmans SA, Vader P, van Dommelen SM, van Solinge WW, Schiffelers RM (2012). Exosome mimetics: a novel class of drug delivery systems. Int J Nanomedicine.

[188] Ramasubramanian L, Kumar P, Wang A (2019). Engineering extracellular vesicles as nanotherapeutics for regenerative medicine. Biomolecules.

