# Supplementary material for: Comprehensive insight into endothelial progenitor cell-derived extracellular vesicles as a promising candidate for disease treatment
Source: Stem Cell Res Ther. 2022 Jun 7;13:238. doi: 10.1186/s13287-022-02921-0 (PMC9172199; doi:10.1186/s13287-022-02921-0)
Supplement: Supplementary file 1 — Additional file1: Table S1. The beneficial effects of EPC-EVs on various diseases. [file 13287_2022_2921_MOESM1_ESM.docx]

**Table S1.** The beneficial effects of EPC-EVs on various diseases

| Disease | Experimental model | EPC source | EPC culture conditions | Characterization of EPC | Surface antigens | EPC-EVs isolation method | Morphology | Size distribution | Method | EVs modification | Proposed mechanism | Refs |
| --- | --- | --- | --- | --- | --- | --- | --- | --- | --- | --- | --- | --- |
| AKI | Male wistar rats acute renal ischemia–reperfusion injury model | Human peripheral blood | / | Staining for rat endothelial cell antigen-1 [RECA-1], FACS | CD34, CD154, a4 and b1 integrin, L-selectin | Ultracentrifugation, 100,000 g for 1 h at 4 °C | TEM | NTA | FACS/WB/ELISA | Knock down miR-126 and miR-296 | MiR-126 and miR-296 have a key role in MV-associated renoprotective effects | [91] |
| AKI | Mice sepsis-induced AKI model. | Human cord blood | EBM-2 | / | CD9, CD63, CD81 | Centrifugation, 12 000 g at 4 C for 70 min | TEM | NTA | WB/ELISA | Knock down miR-93-5p | EPC-EVs secreting miR-93-5p and silencing KDM6B reduce vascular leakage and organ injury via the regulation of the KDM6B/H3K27me3/TNF-α axis | [94] |
| AKI | Caecal ligation and puncture-induced AKI rats model | Rat bone marrow | EGM-2, plates precoated with human fibronectin | Stained with Dil-ac-LDL and UEA-1, IF | CD9, CD63, CD81 | Centrifugation, 10 000 g at 4 C for 1 h | TEM | / | WB/IF | Knock down miR-21-5p | EPC-EVs derived miR-21-5p can alleviate the kidney injury caused by sepsis by downregulating RUNX1 | [95] |
| Glomerulonephritis | Rats glomerulonephritis model | Human peripheral blood | / | Staining for rat endothelial cell antigen-1 [RECA-1], FACS, IF | Factor H, CD55, CD59 | EV isolation kit | TEM | NTA | IF/ELISA/FACS/WB | / | / | [97] |
| Glomerulonephritis | Complement and cytokine-mediated models of human glomerular endothelial cells and podocytes injury | Human peripheral blood | EGM-2, plates precoated with human fibronectin | FACS/IF | / | Ultracentrifugation, 100,000 g for 1 h at 4 °C | TEM | NTA | FACS/ELISA/IF | / | / | [98] |
| ALI | ALI rats model induced by the lipopolysaccharide | Rat bone marrow | EGM-2, plates precoated with human fibronectin | Stained with Dil-ac-LDL and UEA-1 | CD63, Alix, TSG101 | Ultracentrifugation, 100,000 × g for 120 minutes at 4°C | TEM | NTA | WB | Knock down miR-126 | MiR-126 in EPC-EVs contribute to pulmonary repair by downregulating SPERD1 and enhancing RAF/ERK signaling pathways | [102] |
| ALI | ALI rats model induced by the lipopolysaccharide | Human cord blood | EBM-2 | / | CD9, CD63, CD81 | EV isolation kit | / | NTA | WB | Knock down miR-126 | EPC-EVs can mitigate lung injury potentially through the delivery of miR-126 to epithelial cells | [103] |
| Bone defect | Unilateral tibial distraction osteogenesis rats model | Rat bone marrow | EGM-2, plates precoated with rat fibronectin | / | CD9, Alix, TSG101 | Ultracentrifugation, 100 000 x g for 70 min at 4 °C | TEM | Tunable resistive pulse sensing [TRPS] | WB/ELISA | Knock down miR-126 | MiR-126 derived from EPC-EVs enhances endothelial cell proliferation, migration and angiogenesis | [106] |
| Bone defect | In vitro bone marrow stromal cells model | Mouse bone marrow | / | / | CD63 | Ultracentrifugation,120,000 x g for 2 h at 4˚C |  |  | WB | / | / | [107] |
| Bone defect | Mice femur fracture model | Mouse umbilical cord blood | EGM‐2 | Stained with Dil | CD63, CD81 | Ultracentrifugation,100 000 × g for 1 hour at 4°C. | TEM | / | WB/ELISA | Knock down miR-124 | EVs induce osteoclastic differentiation through the lncRNA MALAT1 associated inhibition of miR-124 | [108] |
| Osteoporosis | Steroid-induced osteoporosis mice model | Mouse bone marrow | EBM-2 | Stained with Dil-ac-LDL and UEA-1 | CD9, CD63, CD81 | EV isolation kit | TEM | NTA | WB/ELISA | / | / | [110] |
| Stroke | Hypoxia/reoxygenation injury model on human brain endothelial cells | Mouse bone marrow | EBM-2 | Stained with double-positive Di-LDL and Bs-Lectin, FCM | CD34, VEGFR2, Annexin V | Ultracentrifugation, 120,000 g for 1 h | TEM | / | WB/ELISA/FCM | / | / | [115] |
| Stroke | Hypoxia/reoxygenation-injured human brain endothelial cells model | Human | Complete growth medium | / | CD63 | Ultracentrifugation, 170, 000 g for 90 mins | / | NTA | WB/ELISA | Overexpress miR-210 | EPC-EVs derive miR-210 to protect H/R-injured endothelial cells by improving mitochondrial function | [116] |
| Stroke | Oxyhemoglobin-induced neuron injury model | Human | Complete growth medium | / | / | Ultracentrifugation, 170,000g for 90 min | / | NTA | WB | Overexpress miR-137 | MiR-137 overexpression boosts the neuroprotective effects of EPC-EVs against apoptosis and mitochondrial dysfunction in oxyhemoglobin-treated SH-SY5Y cells | [117] |
| ALS vascular injury | Rat brain endothelial cells model exposed to ALS mouse plasma | Human bone marrow | / | / | / | EV isolation kit | / | NTA | / | / | / | [121] |
| Myocardial fibrosis | Human cardiac fibroblasts [CFs] | Human peripheral blood | / | Stained with Dil-ac-LDL and UEA-1, IF, FCM | CD63, TSG101, HSP70 | Ultracentrifugation, 100,000 g for 90min at 4 C | / | / | IF/WB/FCM | / | / | [124] |
| Myocardial infarction | Rats model of myocardial infarction induced by left anterior descending ligation | Human peripheral blood | EGM-2 | Stained with Dil-ac-LDL and UEA-1, IF | Alix, CD63, TSG101 | Ultracentrifugation, 100,000 g for 70mins at 4°C | / | / | IF/WB | Knock down miR-218-5p and miR-363-3p | Exosomal miR-218-5p/miR-363-3p from EPCs ameliorate myocardial infarction by targeting the p53/JMY signaling pathway | [125] |
| Myocardial infarction | Rats model of myocardial infarction induced by left anterior descending coronary artery | Human peripheral blood | EGM-2 | Stained with Dil-ac-LDL and UEA-1, IF | Alix, CD63, TSG101 | Ultracentrifugation, 100,000 g for 70 min | / | / | WB/IF/ELISA | Overexpress miR-1246 and miR-1290 | MiR-1246 and miR-1290 in EPC-EVs are both beneficial for angiogenesis in MI by inducing upregulation of ELF5 and SP1 | [126] |
| Myocardial infarction | Mice model of acute myocardial infarction | Rat bone marrow | / | / | / | Ultracentrifugation, 100,000 g for 1 h | / | NTA | WB | Knock down Integrin-Linked Kinase | ILK knockdown rescued IL-10 deficiency/inflammation- induced EPC exosomes dysfunction in myocardial repair | [127] |
| Myocardial infarction | Rats model of myocardial infarction induced by left anterior descending ligation | Rat bone marrow | / | FCM | Alix, CD63, CD9 | EV isolation kit | TEM | / | WB/FCM | Overexpress miR-144-3p | Exosomal miR-144-3p disturbed the MMP-9 pathway by inhibiting Ets1 expression in MSCs, and subsequently impaired the mobilization of EPCs from bone marrow microenvironment | [128] |
| Myocardial infarction | Rats Model of Myocardial Infarction | Rat bone marrow | EGM-2 | / | / | EV isolation kit | TEM | NTA | / | / | / | [131] |
| Myocardial infarction | Rats Model of Myocardial Infarction | rat bone marrow | EGM-2 | Stained with Dil | / | EV isolation kit | / | / | / | / | / | [132] |
| Vascular injury | Rats balloon-induced vascular injury model | Human umbilical cord blood | EGM-2, plates pre-coated with type I rat tail collagen | Stained with Dil-ac-LDL and UEA-1 | CD9, CD63, CD81 | Ultracentrifugation, 100,000g for 1 h at 4°C | TEM | / | WB | / | / | [134] |
| Carotid artery injury | Rats carotid artery balloon injury model | Human fetal aorta | EGM-2 | Stained with Dil-ac-LDL | CD9, CD63, CD81 | EV isolation kit | TEM | / | WB | / | / | [136] |
| Vascular injury | Rats model of balloon-induced vascular injury | Human peripheral blood and cord blood | EBM-2 | / | CD9, CD63, CD81 | Ultracentrifugation, 100,000 × g for 120 minutes | TEM | NTA | WB | / | / | [137] |
| Vascular injury | Rats model of balloon-induced vascular injury | Umbilical cord blood | EGM-2 | Stained with Dil-ac-LDL and UEA-1 | CD9, CD63, CD81 | Ultracentrifugation, 100,000×g for 120 min | TEM | NTA | WB | Knock down miR-21-5p | MiR-21-5p inhibits THBS1 expression and promotes vascular endothelial cell proliferation, migration and tube formation | [138] |
| Vascular injury | Rat aorta endothelial cells model with downregulated GPRC6A | Rat | / | IF | CD9, CD81 | EV isolation kit | TEM | NTA | WB/IF | Downregulate GPRC6A | EPC-EVs derive osteocalcin to participate in the increase of endothelial cell proliferation, migration, NO formation, and angiogenesis | [140] |
| Vascular injury | Ang II-induced endothelial cells injury model | Human | Complete growth medium | / | / | Ultracentrifugation, 170,000 g | / | / | WB/ELISA | / | / | [142] |
| Vascular injury | Hyperoxia injury pulmonary microvascular endothelial cells model | Rat bone marrow | EGM-2 | Stained with Dil-ac-LDL and UEA-1 | TSG101, CD63 | EV isolation kit | TEM | NTA | WB | / | / | [145] |
| Atherosclerosis | Atherosclerotic mice model | Mouse umbilical cord blood | EBM-2 | IF | CD9, CD81, Alix | Ultracentrifugation, 110,000 × g at 4 °C for 70 min | TEM | NTA | WB/IF/ELISA | Knock down miR-199a-3p | EPC-EVs inhibited ferroptosis of endothelial cells via the miR-199a-3p/SP1 axis and ultimately alleviated atherosclerosis | [146] |
| Sepsis | Murine Model of Sepsis | Human cord blood | EBM-2 | / | / | EV isolation kit | / | NTA | WB/ELISA | Knock down miR-126-5p and 3p | EPC-EVs prevent microvascular dysfunction and improve sepsis outcomes potentially through the delivery of miR-126 | [149] |
| Sepsis | Rats model of sepsis | Rat bone marrow | EBM-2 | Stained with Dil-ac-LDL and UEA-1, FCM | CD9, CD63, CD81 | EV isolation kit | TEM | NTA | WB/ELISA/FCM | Knock down miR-375-3p | MiR-375-3p activates BRD4-mediated PI3K/AKT pathway to ameliorate myocardial injury in rats with sepsis | [150] |
| Sepsis | Caecal ligation and puncture [CLP]-induced septic mice model | Mouse umbilical cord blood | / | IF | ALIX, TSG101, CD9 | Ultracentrifugation | TEM | NTA | WB/ELISA/IF | Upregulate TUG1 | EPC-EVs containing TUG1 upregulates SIRT1 expression by binding to miR-9-5p to promote macrophages M2 polarization | [153] |
| Diabetes | Rats model of type 1 diabetes | Human umbilical cord blood | EGM-2, plates pre-coated with type I rat tail collagen | Stained with Dil-ac-LDL and UEA-1, FCM | CD9, CD63, CD81 | Ultracentrifugation, 100,000×g for one hour at 4 °C | / | / | WB/FCM | / | / | [157] |
| Diabetes | Diabetic rats skin wound model | Human umbilical cord blood | EGM-2 | Stained with Dil-ac-LDL and UEA-1, IF, FCM | CD9, CD63, CD81 | Ultracentrifugation, 100,000× g for one hour | TEM | Tunable resistive pulse sensing [TRPS] | WB/IF/FCM | Inhibit ERK1/2 signaling | Erk1/2 signaling mediated the EPC-EVs-induced pro-angiogenic effects on endothelial cells | [158] |
| Diabetes | Diabetic mice skin wound model | Mouse bone marrow | EGM-2, plates precoated with human fibronectin | IF | / | EV isolation kit | TEM | / | IF | Knock down miRNA-221-3p | EPC-EVs derive miRNA-221-3p to promote skin wound healing in normal and diabetic mice | [159] |
| Diabetic ischemic stroke | Type II diabetic mice | Mouse bone marrow | / | Stained with Dil-ac-LDL and Bs-Lectin, IF | / | Ultracentrifugation, 100 000 g for 90 minutes at 4°C | / | NTA | WB/IF | Overexpress miR-126 | MiR-126 in EPC-EVs cure diabetic ischemic stroke by promoting neurological functional recovery via accelerating angiogenesis and neurogenesis | [162] |
| Diabetes | Mice model of diabetic atherosclerosis | Mouse bone marrow | EBM-2 | / | CD63, CD81 | EV isolation kit | TEM | / | ELISA | / | / | [164] |
