# Supplementary material for: Comprehensive insight into endothelial progenitor cell-derived extracellular vesicles as a promising candidate for disease treatment
Source: Stem Cell Res Ther. 2022 Jun 7;13:238. doi: 10.1186/s13287-022-02921-0 (PMC9172199; doi:10.1186/s13287-022-02921-0)
Supplement: Supplementary file 2 — Additional file2: Table S2. The beneficial effects of ADSC- and BMSC-EVs on various diseases. [file 13287_2022_2921_MOESM2_ESM.docx]

| **Table S2.** The beneficial effects of ADSC- and BMSC-EVs on various diseases | | | | | | | | | | | | |
| --- | --- | --- | --- | --- | --- | --- | --- | --- | --- | --- | --- | --- |
| Disease | Experimental model | Stem cell source | Stem cell culture condition | Characterization of stem cell | Surface antigens | EV isolation method | Morphology | Size distribution | Method | EVs modification | Proposed mechanism | Refs |
| Osteoporosis | Osteocyte-like cell line MLO-Y4 apoptosis induced by hypoxia and serum deprivation | Mice adipose tissue | DMEM/F12 | Green Fluorescent | CD9, CD63, and HSP70 | 110000 g for 70 min at 4 C | TEM | NTA | WB | / | The antiapoptotic effects of ADSCs-EVs were attained through the upregulation of Bcl-2/Bax, the suppression of ROS, cytochrome c generation and caspase-3, caspase-9 activation. | 30473217 |
| Nerve injury | Acute sciatic nerve injury induced adult wistar rats | Rats adipose tissue | DMEM/F12 | IF | CD9, CD63 | Isolation kit | / | / | WB/IF/FCM | / | ADSC-EVs increase neurite outgrowth in vitro and enhance regeneration after sciatic nerve injury in vivo. | 29931510 |
| Stroke | Rats model of Middle Cerebral Artery Occlusion | Rats adipose tissue | DMEM | IF | / | Isolation kit | / | / | WB/IF/ELISA | Overexpress miR-126 | ADSC-EVs derive miR-126 post stroke improve functional recovery, enhance neurogenesis, inhibit neuroinflammation | 30899379 |
| Stroke | Rats model of Middle Cerebral Artery Occlusion | Rats adipose tissue | DMEM/F12 | / | CD9, CD63, and Alix | Isolation kit | / | / | WB/FCM | Overexpress miR-181b-5p and miR-212-5p | ADSC-EVs promote the angiogenesis of brain microvascular endothelial cells after oxygen-glucose deprivation via miR-181b-5p/TRPM7 axis | 29705934 |
| Cerebral ischemia | Rat model of focal cerebral I/R injury | Human or rats adipose tissue | EGM-2 | fluorescein isothiocyanate (FITC-F) or phycoerythrin (PE) | CD63, CD81, and TSG101. | Isolation kit | TEM | / | WB/FCM | PEDF overexpression | PEDF-modified ADSCs-EVs ameliorated cerebral ischemia–reperfusion injury by regulating autophagy and apoptosis | 30142325 |
| Breast cancer | Breast cancer cell line MCF7 | Human adipose tissue | DMEM/F12 | Dil | CD63, and HSP70 | 100,000 g for 1 h at 4 C | TEM | / | WB | / | ADSC-Evs promoted MCF7 migration through the activation of the Wnt signaling pathway | 23812844 |
| Hepatocellular Carcinoma | Rat model of hepatocellular carcinoma | Rats adipose tissue | DMEM | Fluorescein isothiocyanate (FITC-F) or phycoerythrin (PE) | CD63,β-catenin and TSG101 | Isolation kit | TEM | / | WB | / | ADSC-EVs promoted NKT-cell antitumor responses in rats, thereby facilitating HCC suppression | 26345219 |
| Wound | HaCaT cells impaired by H2O2 | Human adipose tissue | DMEM/F12 | / | CD9, CD63 | Isolation kit | TEM | / | WB/FCM | / | ADSC‐EVs activates Wnt/β‐catenin signaling to prompt wound healing | 30681184 |
| Wound | Mice model of wound | Human adipose tissue | DMEM/F12 | Oil red O and 63Alizarin Red S | CD63, HSP70 | 100,000 g for 70 min at 4 °C | TEM | / | WB/ELISA/FCM | / | ADSC-EVs promote proliferation and intracellular collagen secretion in human dermal fibroblasts via the PI3K/Akt signaling pathway | 29964051 |
| Scar | Mice model of skin wound model | Human adipose tissue | DMEM/F12 | IF | CD9, CD63 | Isolation kit | / | NTA | WB/IF/FCM | / | ADSC-EVs promote ECM reconstruction in cutaneous wound repair | 29042658 |
| Alzheimer's disease | In vitro AD model | Human adipose tissue | EGM-2 | / | HSP70, CD63, and CD9 | Isolation kit | / | / | ELISA/WB/FCM | / | ADSC-EVs reduces these pathological phenotypes of the AD in vitro model and augment neurite outgrowth of AD cells | 29625119 |
| Huntington’s disease | In vitro HD model | Human adipose tissue | EGM-2 | Em48 (red) or DAPI (blue)-stained | / | Isolation kit | / | / | WB | / | ADSC-EVs have a therapeutic potential for treating HD by modulating representative cellular phenotypes of HD. | 27177616 |
| Amyotrophic lateral sclerosis | In vitro ALS model | Human adipose tissue | EGM-2 | Cytoplasm (red) and DAPI staining (blue) | HSP70, CD63 and CD9 | Isolation kit | / | / | WB | / | ADSC-EVs modulates cellular phenotypes of ALS including SOD-1 aggregation and mitochondrial dysfunction, | 27641665 |
| AKI | Rats model of AKI | Rats adipose tissue | DMEM | IF | CD63,β-catenin and TSG101 | Isolation kit | / | / | WB/IF | / | ADSC-Evs protect the kidney from IR injury | 27156061 |
| Myocardial infarction | Rats model of myocardial infarction | Rats adipose tissue | DMEM | / | CD9, CD63, CD81, HSP70 | 100,000 g for 1 h | TEM | / | WB/FCM/ELISA | / | ADSC-EVs can protect ischemic myocardium from I/R injury through activating the Wnt/β-catenin signaling pathway by exerting the  anti-apoptotic and pro-survival effects on cardiomyocytes | 28582278 |
| Myocardial infarction | Rats model of myocardial infarction | Rats adipose tissue | DMEM | Fluorescein isothiocyanate (FITC-F) or phycoerythrin (PE) | CD63, CD9, and TSG101 | Isolation kit | TEM | NTA | WB | Overexpress miR-126 | The expression of miR-126-enhanced ADSC-EVs can prevent myocardial damage | 29241208 |
| Premature ovarian insufficiency | Mice model of Premature ovarian insufficiency | Human adipose tissue | DMEM/F12 | / | CD63, CD9, and CD81 | Isolation kit | TEM | / | FACS/WB/ELISA | / | ADSC-EVs recover the ovarian function of POI by upregulating SMAD expression | 30092819 |
| Liver fibrosis | CCl4-induced liver fibrosis mice model | Human adipose tissue | DMEM | IF | CD63, CD81 | Isolation kit | TEM | / | WB/IF/ELISA | Overexpress miR-181-5p | ADSC-EVs derived miR-181-5p prevent liver fibrosis via autophagy activation | 28382720 |
| Nerve injury | Rats model of sciatic nerve injury | Rats bone marrow | DMEM | / | / | 100,000 g for 1 h at 4°C | TEM | / | FCM/FACS | / | BMSC-EVs could restore nerve function and promote nerve regeneration in sciatic nerve crush injury model | 31966893 |
| Stroke | Rats model of middle Cerebral Artery Occlusion | Rats bone marrow | MEM | / | / | 100,000 g for 1 h at 4°C | / | / | / | / | BMSC-Evs improves functional recovery and enhances neurite remodeling, neurogenesis, and angiogenesis | 23963371 |
| Traumatic brain injury | Rats model of TBI | Rats bone marrow | MEM | IF | / | Isolation kit | TEM | / | WB | / | BMSC-EVs effectively improve functional recovery, by promoting endogenous angiogenesis and neurogenesis and by reducing inflammation in rats after TBI. | 25594326 |
| Spinal cord injury | Rats model of SCI | Rats bone marrow | DMEM | IF | CD9, CD63, and CD81 | 110,000×g for 70 min | TEM | / | WB/IF | / | BMSC-EVs can effectively inhibit the migration of pericytes, thereby maintaining the integrity of the BSCB after SCI | 30914918 |
| Spinal cord injury | Rats model of SCI | Rats bone marrow | DMEM/F-12 | FCM | CD9, CD63, and CD81 | 100,000 g for 6 h at 4℃ | TEM | / | WB/FCM | / | BMSC-EVs reduce tissue damage, improve functional recovery, and inhibit neural cell apoptosis after SCI by activating the Wnt/b-catenin signaling pathway | 31423807 |
| Spinal cord injury | Rats model of SCI | Rats bone marrow | DMEM | IF | CD9, CD63, and CD81 | Isolation kit | / | / | WB/IF | Overexpress miR-133b | BMSC-EVs derived miR-133b preserved neurons, promoted the regeneration of axons, and improved the recovery of hindlimb locomotor function following SCI | 30524227 |
| Multiple sclerosis | Experimental autoimmune encephalomyelitis (EAE) mouse model | Human bone marrow | MEM | IF/FCM | CD81, HSP70, TSG101 | 120,000 × g for 2.5 h at 4 °C | TEM | NTA | WB/FCM/IF | / | BMSC-EVs can potentially serve as cell-free therapies in creating a tolerogenic immune response to treat autoimmune and central nervous system disorders | 31117376 |
| Myocardial infarction | Rats model of AMI | Rats bone marrow | IMDM | FCM/IF | Alix, TSG101, CD63, and CD81 | 120,000g for 70 min | TEM | NTA | WB/FCM/ELISA/IF | / | Combination of BMSC-Evs and BMSCs reduces scar size and restores heart function after AMI | 31601262 |
| Myocardial infarction | Rats model of AMI | Human bone marrow | MEM | FCM/IF | CD63, CD81 | 100,000×g for 1 h at 4 °C | / | / | WB/FCM/IF | / | BMSC-EVs can protect cardiac tissue from ischemic injury at least by means of promoting blood vessel formation | 24337504 |
| Myocardial infarction | Mice model of AMI | Mice bone marrow | α-MEM | FCM | Alix, TSG101 | 140,000 ×g for 90 min at 4 ℃ | TEM | Light scattering microscopy | WB/FCM | Overexpress miR-125b-5p | Hypo-BMSC-EVs facilitate ischemic heart repair via the antiapoptotic miR-125b-5p. | 30613290 |
| Myocardial infarction | Mice model of myocardial infarction | Mice bone marrow | DMEM | FCM | CD9, CD63, and Alix | Repeated ultracentrifugation | TEM | NTA | WB/FCM | Overexpress miR-210 | Hypoxic culture enhances the activity of Mir-210 and nSMase2 in BMSC-EVS, which is at least partly responsible for the enhanced cardioprotective effect of exosomes in hypoxic-treated cells. | 29141446 |
| Bone defect | Rats model of defects | Human bone marrow | α-MEM | FCM/IF | CD63 | 120 000 g for 120min | / | / | WB/FCM | / | BMSC-EVs substantially promoted bone regeneration in SD rats with calvarial defects. | 26911789 |
| Bone defect | Rats model of calvarial defects | Human bone marrow | MEM | IF | CD9, CD63, TSG101, and GM130 | 110,000×g for 70 min at 4 °C | TEM | / | WB/IF | / | Dimethyloxaloylglycine-stimulated BMSC-EVs promote neovascularization via the AKT/mTOR pathway and enhance bone regeneration in critical-sized bone defects in rats | 31747933 |
| Bone defect | Mice model of fracture | Human bone marrow | DMEM | / | CD9, CD81 | 70 minutes at 180,000g at 4°C | TEM | / | WB | / | BMSC-EVs play an important role in fracture healing through facilitation of endochondral ossification. | 27460850 |
| Bone defect | Mice model of fracture | Rat bone marrow | DMEM | FCM | / | 20,000× g for 1h at 4 °C | SEM | Confocal microscope | FCM | / | BMSC-EVs-modified scaffolds promote vascularization in the grafts, thereby enhancing bone regeneration. | 28367979 |
| Osteoarthritis | In vitro osteoarthritic chondrocytes | Human bone marrow | DMEM | IF | CD9, CD63 | 100,000 × g for 16 h | TEM | NTA | WB/IF | / | BMSC-EVs have both regenerative and immunoregulatory properties in human OA cartilage. | 29463990 |
| Osteoarthritis | In vitro in a human MSC model | Human bone marrow | α-MEM | FCM/IF | CD9, CD63, CD81, and HSP70 | 100,000 × g for 1 h | TEM | / | WB/IF/FCM | Overexpress miR-92a-3p | BMSC-EVs miR-92a-3p is efficacious in cartilage development and degeneration. | 30257711 |
| AKI | Mice model of AKI | Human bone marrow | MSCBM | Phycoerythrin (PE) or FITC | / | 100,000 g for 1 h at 4 °C | / | / | FACS/WB/ELISA | / | BMSC-EVs may activate a proliferative program in surviving tubular cells after injury via a horizontal transfer of mRNA. | 19389847 |
| AKI | Mice model of AKI | Human bone marrow | DMEM | IF | CD63 and CD9 | 100.000g for 1 hour at 4°C | TEM | / | WB/IF | / | BMSC-EVs recruited to the site of injury may reprogram the damaged cells stimulating their proliferation and favouring tissue regeneration through the contribution of growth factors and the transfer of genetic material | 23082760 |
| Skeletal muscle defect | Mice model of cardiotoxin-induced muscle injury | Human bone marrow | DMEM | IF | CD9, CD81 | 70 min at 110,000 ´ g | / | Tunable resistive pulse sensor | WB/ELISA/IF | Overexpress miR-494 | BMSC-EVs derived miR-494 promote muscle regeneration by enhancing myogenesis and angiogenesis | 25862500 |
| Tendon injury | Rat patellar tendon defect model | Rats bone marrow | DMEM | FCM/IF | CD9, Alix, TSG101 | 100,000 g for 70 min at 4°C | TEM | NTA | WB/FCM/IF | / | BMSC-EVs promote tendon regeneration by facilitating the proliferation and migration of endogenous tendon stem/progenitor cells | 32027991 |
| Wound | In vitro wound fibroblasts | Human bone marrow | α-MEM | Dil and DiO | CD9, CD81, CD63, Hsp70, Alix, Flotillin-1 | 100,000 g for 70 minutes at 4°C | TEM | / | WB | / | BMSC-EVs enhance the growth and migration of normal and chronic wound fibroblasts, and induce angiogenesis in vitro | 25867197 |
| ALI | Pigs model of ALI | Femur bone marrow | DMEM | FCM/IF | CD9, CD63, and CD81 | 25,000 rpm for 70 min at 4 °C | TEM | / | WB/FCM/IF | / | BMSC-EVs attenuate influenza virus-induced acute lung injury | 29378639 |
| ALI | Mice model of fixed pressure hemorrhagic shock | Human bone marrow | EGM-2 | FCM | CD9, CD63, and CD81 | / | / | / | FCM | / | BMSC-EVs modulate cytoskeletal signaling and attenuate lung vascular permeability after hemorrhagic shock. | 29251710 |
| Ulcerative colitis | Mice model of ulcerative colitis | Mice bone marrow | DMEM | IF | CD63, CD81, and TSG101 | 100,000 ×g for 70 min | TEM | / | ELISA/WB/IF | / | BMSC-EVs promote M2-like macrophage polarization and relieve inflammatory responses, in turn attenuating DSS-induced UC | 31005036 |
| Intervertebral disc degeneration | In vitro nucleus pulposus cells | Human bone marrow | DMEM/F-12 | Fluorescence confocal microscope | CD63 and TSG101 | 100,000 × g for 70 min | TEM | / | WB | / | BMSC-EVs promote nucleus pulposus cells proliferation and healthier extracellular matrix production in the degenerate nucleus pulposus cells | 28486958 |
| Alzheimer’s disease | Mice model of AD | Mice bone marrow | DMEM | IF | CD9 and CD63 | Isolation kit | TEM | / | WB/ELISA/IF | / | BMSC-EVs reduce Aβ deposition and improve cognitive function recovery in mice with Alzheimer’s disease by activating sphingosine kinase/sphingosine-1-phosphate signaling pathway | 33300254 |
| Acute liver failure | In vitro D-GaIN/LPS-induced hepatocyte apoptosis | Human bone marrow | DMEM | FCM | CD9, CD63 and CD81 | 100,000× g for 1 hr at 4 °C | TEM | NTA | WB/FCM | / | BMSC-EVs attenuate D-GaIN/LPS-induced hepatocyte apoptosis by activating autophagy in vitro | 31695322 |
| Sepsis | Mice model of sepsis induced by cecal ligation and puncture (CLP) | Mice bone marrow | DMEM | IF | CD63, CD81 | 13,000 rpm for 30min at 4°C | / | / | WB/ELISA/IF | Knock down miR-223 | BMSC-EVs have cardio-protection in sepsis via miR-223 | 26348153 |
